# Genome-Resolved Metatranscriptomics Provide Insights on Immigration Influence in Structuring Microbial Community Assembly of a Full-Scale Aerobic Granular Sludge Plant

**DOI:** 10.1021/acs.est.4c14471

**Published:** 2025-03-19

**Authors:** A. Y. A. Mohamed, Laurence Gill, Alejandro Monleon, Mario Pronk, Mark van Loosdrecht, Pascal E. Saikaly, Muhammad Ali

**Affiliations:** † Department of Civil, Structural & Environmental Engineering, Trinity College Dublin, The University of Dublin, Dublin D2, Ireland; ‡ Department of Biotechnology, Delft University of Technology, Delft 2629 HZ, The Netherlands; § Environmental Science and Engineering Program, Biological and Environmental Science and Engineering (BESE) Division, King Abdullah University of Science and Technology (KAUST), Thuwal 23955-6900, Saudi Arabia; ∥ Water Desalination and Reuse Center, Biological and Environmental Science and Engineering (BESE) Division, King Abdullah University of Science and Technology (KAUST), Thuwal 23955-6900, Saudi Arabia

**Keywords:** genome-resolved metatranscriptomics, aerobic granular sludge, immigration, community assembly, biological wastewater treatment

## Abstract

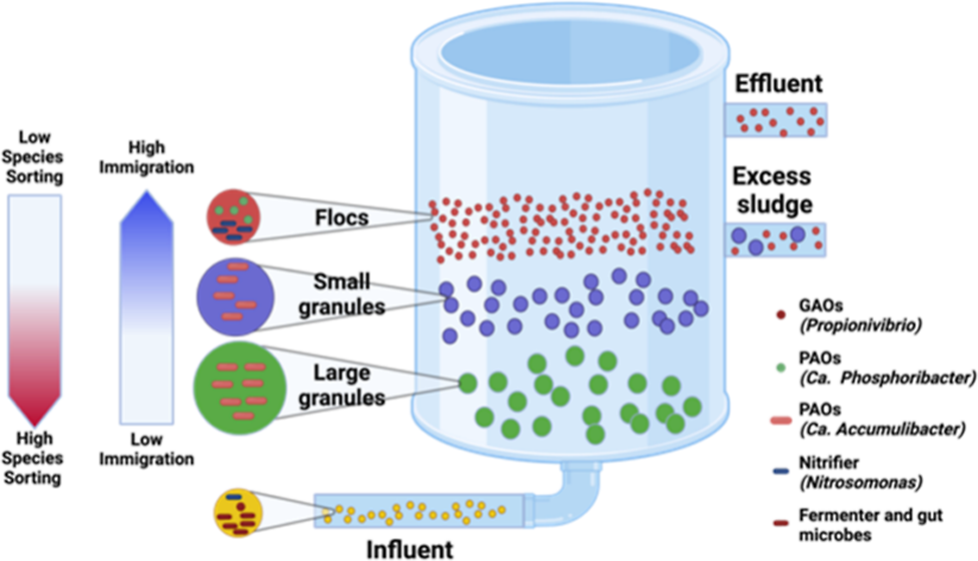

Understanding the relative influence of immigration and species sorting in wastewater treatment systems is essential, as bacteria in influent wastewater can significantly impact treatment system functionality. This study investigated the contribution of immigration to the community assembly of different-sized microbial aggregates in a full-scale aerobic granular sludge (AGS) system using genome-resolved metatranscriptomics. Our novel analysis revealed that negative-net-growth-rate populations, which persist due to immigration, can exhibit substantial activity and potentially contribute to the AGS system’s functionality. The results also highlighted that sulfate-reducing and fermenting bacteria, along with some nitrifiers and glycogen-accumulating organisms (GAOs), were more active in the influent wastewater, serving as a continuous source of both beneficial and competing immigrants to the AGS system. Granular sludge (size >0.2 mm) demonstrated a robust capacity to resist immigration effects from competing immigrants, whereas flocculent sludge (size <0.2 mm) was more susceptible. Importantly, flocculent sludge harbored functional microbial groups such as active nitrifiers and fermentative polyphosphate-accumulating organisms (PAOs) belonging to *Ca. Phosphoribacter*, while granular sludge enriched for active conventional PAOs such as *Ca. Accumulibacter*. These findings provide valuable insights for engineers to design and operate AGS systems by optimizing microbial aggregate sizes and emphasizing the importance of influent microbial characterization in the design of wastewater treatment plants to enhance the functionality and activity of AGS systems.

## Abstract 2

Wastewater is a source of both beneficial and competing immigrants. This study revealed that negative-net-growth-rate populations, which persist due to wastewater immigration, can exhibit substantial activity, and potentially contribute to the system’s functionality.

## 1 Introduction

The aerobic granular sludge (AGS) wastewater treatment process was invented around the year 2000 and has gained significant attention due to its superior nutrient removal performance, lower (40%) energy consumption, and smaller (75%) footprint when compared to conventional activated sludge (CAS) systems.^1−3^ In the AGS system, different-sized microbial aggregates (flocs have typical size <0.2 mm and granules have typical size >0.2 mm) coexist in the same reactor and exhibit distinct microbial community structure and metabolic function influenced by factors such as substrate availability, oxygen gradients, and nutrient transport limitations.^4−6^ Local factors, such as operational parameters [e.g., chemical oxygen demand (COD) fractionation, pH, temperature, salinity, and solid retention time (SRT)] are considered deterministic parameters and play a significant role in shaping microbial communities of different-sized microbial aggregates in the AGS system.^7−9^ Different-sized microbial aggregates coexisting in the same tank have different SRTs, yet they are exposed to the same microbial dispersal rate from influent wastewater. This renders AGS as a unique engineered ecosystem to study the roles of local (species sorting) and regional (immigration) factors responsible for shaping the bacterial community assembly. The degree to which the assembly of local communities in engineered ecosystems is influenced by either local factors, often referred to as “species sorting”, or regional factors linked to dispersal, which dictate the influx of immigrants from the source habitat to the local community (sink habitat), is consistently a subject of uncertainty.^10,11^

Recently, mass balance coupled with 16S rRNA (16S rRNA) amplicon sequencing has been successfully implemented to effectively quantify the rate of bacterial immigration, enabling the differentiation between positive- and negative-net-growth-rate species.^6,12,13^ Consequently, this approach helps pinpoint the bacteria that are likely of significance for the functioning of systems.^6,12,13^ It was found that the structuring of the microbial community in the bioreactor compartment of CAS and AGS systems is strongly impacted by local factors (species sorting) rather than regional factors of immigration, with just 10% of the total microbial community in the bioreactor belonging to negative-net-growth-rate taxa, which are present due to mass immigration from the influent.^6^ Further, the immigration rate was significantly higher for smaller sized aggregates, which had shorter SRT, i.e., flocs (FL; 12% of the total reads in AGS system) and small granules (SG; 7%), than for larger-sized aggregates with longer SRT (large granules (LG); 2%).^6^

The above-mentioned studies highlighted that most of the bacteria immigrating from the influent to the bioreactor were decaying. This was evident from the negative values of growth rates, suggesting that they may have little functional role in the system. Nevertheless, it is not sufficient to use the combined amplicon sequencing-mass balance model as a sole indicator for microbial growth or activity of immigrants.^14^ In fact, one could imagine a situation where the immigrant populations have a negative net-growth rate and be fully metabolically active because they are growing at their intrinsic maximum growth rate. Mei et al.^15^ and Matar et al.^16^ adopted a method of using the ratio of 16S rRNA and 16S rDNA (rRNA/rDNA) for individual populations and identified active populations in a full-scale anaerobic digester and membrane bioreactor, respectively. However, the rRNA/rDNA ratio approach failed to generate consistent results compared to using the mass balance approach as the rRNA/rDNA ratio approach underestimates the full potential activity of the microorganism and therefore has a limitation in terms of its estimate of microbial activity. In addition, the 16S rRNA amplicon-based analysis has several biases related to library preparation and could not always classify microbes to a lower taxonomic level, such as genus or species.^17,18^

Therefore, in this study, the relative contribution of immigration in community assembly of different-sized microbial aggregates in a full-scale AGS system was quantified using genome-resolved metagenomics- and genome-resolved metatranscriptomics-based mass balance approaches. Furthermore, differential expression analysis (DESeq2) and relative activity (RNA/DNA) were utilized to define the metabolic status of immigrants. These approaches will help us to better understand and quantify the relative contribution of immigration in the AGS system. Our novel analysis revealed that negative-net-growth-rate populations, which persist due to immigration, can exhibit substantial activity and potentially contribute to the functionality of the AGS system.

## 2 Materials and Methods

### 2.1 Sampling of Full-Scale AGS Treatment Plant

Ringsend Wastewater Treatment Plant (WWTP) is located in Dublin, Ireland, and it serves about 1.7 million population equivalents (PE), approximately one-third of the Irish population (Figure S1, Appendix A). The AGS system, based on the Nereda technology owned by Royal HaskoningDHV, was retrofitted into the treatment plant in 2017 to increase its capacity to 2.4 million PE, making it one of the largest AGS plants in the world. There are 24 pre-existing sequencing batch reactor (SBR) tanks, of which 8 were transformed into AGS. In addition, 6 new AGS reactors were constructed on the remaining available footprint. Each SBR tank has a volume of 13,993 m^3^. The current study focused on sampling a single AGS reactor under the assumption that other AGS reactors exhibit similar characteristics. Grab samples (300 mL) were collected in triplicate (*n* = 3) once per week over three consecutive weeks (15, 22, and 29 June, 2022) from the influent, the AGS reactor, the effluent, and the excess sludge. Influent samples were collected after the equalization tank, immediately before entering the AGS system. These tanks provide an effective buffering capacity, ensuring that the influent samples are representative and reflect the average daily wastewater characteristics. Samples from the AGS reactor were taken from the bottom of the tank (at a depth of 4 m) during both anaerobic (feeding) and aerobic phases after 30 min from the start of each phase to ensure good onset of microbial activity. Excess sludge samples were collected from the sludge holding tank (total capacity: 7790 m^3^), which ensures homogenized and representative samples. Effluent samples were collected from effluent gutters. All samples were preserved immediately with RNAlater (Sigma-Aldrich, Merck, USA) to preserve cellular RNA materials from degradation. The samples were then shipped on ice to the lab and kept at 4 °C for 24 h. The mixed liquor suspended solids (MLSS) and excess sludge samples were further sieved and separated into 3 categories: LG (size >1 mm), SG (size 1–0.2 mm), and flocs (FL, size <0.2 mm). Then, all samples including influent, and effluent were centrifuged at 8000 rpm for 10 min in order to obtain a pellet in which RNAlater (Sigma-Aldrich, Merck, USA) was added at an equal volume ratio (1:1), and the samples were kept at −80 °C until DNA and RNA extraction.

### 2.2 Metagenomics Workflow

The RNAlater was removed from the samples prior to DNA extraction, and the pellets were washed several times with a phosphate buffer solution. Total genomic DNA was extracted from triplicate samples (n = 3 each) of influent, AGS (aerobic MLSS only: FL, SG, LG), effluent, and excess sludge using FastDNA SPIN Kit for Soil (MP Biomedicals, Santa Ana, CA, USA) following the manufacturer’s recommendations. The short reads library was constructed for all extracted DNA samples (*n* = 6 × 3) with the VAHTS Universal Plus DNA Library Prep Kit for Illumina according to the manufacturer’s instructions (see Supporting Information: Appendix A). Sequencing of the metagenomics libraries was then performed on the Illumina NovaSeq 6000 platform, using paired-end 150 bp (PE150) sequencing, targeting a sequencing depth of 10 Gb per sample. The long-read DNA libraries were prepared for samples from FL, SG, LG, and excess sludge (*n* = 1 each) using the barcoded SQK_LSK109 DNA library preparation kit (Oxford Nanopore Technologies, Oxford, United Kingdom), following the manufacturer’s protocol (see Supporting Information: Appendix A). The barcoded DNA library was loaded onto primed FLO-PRO002 flow cells and sequenced on a PromethION 48 device (10Gb/Sample). Signal data was base called and demultiplexed with Guppy v. 6.3.9 (Oxford Nanopore Technologies, Oxford, United Kingdom).

Illumina short reads were quality-assessed using FastQC-v0.11.9^19^ and quality filtered using Cutadapt v4.3.^20^ Filtered forward and reserve reads were first concatenated for all samples and then assembled (pooled assembly strategy) using MEGAHIT v1.2.9^21^ to generate contigs, and contigs <1500 bp were eliminated. Following de novo assembly of short reads, contigs were subjected to automated unsupervised binning using VAMB v. 4.1.3,^22^ MaxBin2 v. 2.2.4,^23^ and MetaBAT2 v. 2.15.^24^ Then, the recovered metagenome-assembled genomes (MAGs) were refined and consolidated by MetaWRAP v1.3.^25^ Nanopore long reads of 4 DNA samples (FL, SG, LG, and excess sludge) were quality assessed using NanoPlot v1.43.0 and quality filtered using Filtlong v0.2.1 and Porechop v0.2.4.^26^ De novo assemblies of concatenated long reads were produced with Flye v. 2.9.5,^27^ and the draft assemblies were subsequently polished once with Racon v.1.4.3^28^ and twice with Medaka v. 2.0.1 using Nanopore data (Oxford Nanopore Technologies, Oxford, United Kingdom). The draft assemblies were then put through a last polishing step with Racon v.1.4.3^28^ using Illumina short reads. Contigs shorter than 1500 bp were removed, and MAGs were recovered and refined using similar binning and refinement steps, as described previously for short-read data. Inter and intra assembly MAG dereplication were conducted using dRep v. 3.4.3^29^ using a 95% average nucleotide identity (ANI) threshold and setting minimum MAG length to 0.5 Mbp, minimum completion to 25%, and maximum contamination to 10% thresholds. MAG completeness and contamination levels were assessed using CheckM2 v. 1.0.1.^30^ MAG relative abundances were calculated from read coverage by mapping short reads to dereplicated MAGs using CoverM v. 0.7.0, setting—min-read-percent-identity to 95% and—min-read-aligned-percent to 90%. Taxonomic classification of MAGs was conducted using GTDB-Tk v2.2.6^31^ with ANI thresholds of ≥95% for species-level resolution. Of the 812 recovered MAGs included in downstream analyses, 240 were classified at the species level, while others were resolved at the genus level based on phylogenetic placement (Appendix B). Functional analyses and heatmaps were restricted to MAGs with completeness >50% (540 MAGs), ensuring reliable insights into the microbial community’s metabolic potential. Biological functions of the genus level taxa were identified based on the MiDAS field guide.^32^ Principle coordinates analysis (PCoA) of overall samples similarity of metagenomics-based community composition was generated from the relative abundances data and visualized using the R-package of ampvis2^33^ in R software v3.3.1.

### 2.3 Metatranscriptomics Workflow

After removing RNAlater, total RNA was extracted from triplicate samples (*n* = 3 of each) of influent, AGS (aerobic and anaerobic MLSS: FL, SG, LG), effluent, and excess sludge, using the TRizol Reagent Kit (Invitrogen, Thermo Fisher Scientific, Oregon, USA) according to the manufacturer’s guidelines. The TruSeq Stranded Total RNA with Illumina Ribo-Zero Plus rRNA Depletion (Illumina, CA, USA) was used to deplete rRNA in total RNA and construct the library of metatranscriptome sequencing according to the manufacturer’s instructions (see Supporting Information). Sequencing libraries of the metatranscriptomics was conducted on an Illumina NovaSeq 6000 platform targeting a sequencing depth of 10 Gb per sample and using paired-end 150 bp (PE150) reads. Generated reads were obtained from the NovaSeq 6000 platform and assessed for quality using FastQC-v0.11.9.^19^ Reads trimming and quality filtering were conducted through Cutadapt v4.3.^20^ The rRNA reads were discarded using SortMeRNA 4.3.6^34^ based on SILVA rRNA gene database.^35^ MAGs relative expression were calculated from reads coverage by mapping RNA short reads to all dereplicated MAGs using CoverM v. 0.7.0, setting—min-read-percent-identity to 95%. The count table of RNA mapped reads for all MAGs was also generated using CoverM v. 0.7.0. The PCoA plot of overall samples similarity of metatranscriptomics-based community composition was generated from the relative expressions data and visualized in the same PCoA plot of the metagenomics data set. Bray–Curtis dissimilarity distance matrix and analysis of similarity (ANOSIM) between samples were performed and visualized using the Vegan package in RStudio (R v3.3.1). Hierarchical clustering (method: average) was performed to construct dendrogram cluster trees of samples. Differential expression analysis was conducted on the MAG’s level by importing the count tables of RNA reads to RStudio (R v3.3.1), processed using the default DESeq2 workflow,^36^ and visualized using the R-package of EnhancedVolcano.^37^ Only MAGs with completeness >50% and contamination <10% were qualified for DESeq2 analysis according to minimum information about a MAG (MIMAG).^38^ Differential expression analysis using DESeq2 was used as a method to define the metabolic status of immigrants by directly comparing the activity of species in the influent to the AGS system.

### 2.4 Mass Balance Calculations

An immigrating bacterium in this study is defined as the bacteria shared between the influent and the AGS system. The ability of species to grow or decline in the AGS system is determined by environmental conditions and deterministic selection. Populations with a negative net-growth rate represent species that are declining or disappearing in the AGS system compared to the influent, likely due to factors such as decay. Their persistent presence in the AGS system is attributed to passive transport and significant immigration from the influent. Conversely, species with a positive net-growth rate are those that grow and exhibit increased populations within the AGS system compared to the influent.

To determine positive- and negative-net-growth-rate populations, we utilized metagenomics- and metatranscriptomics-based mass balance approaches similar to the amplicon sequencing-mass balance model.^6,12^ The mass balance calculations were conducted for one AGS reactor. Design and operational data for the AGS plant required for the calculations, including information about plant design and influent, effluent, and sludge characteristics, are presented in Tables S1 and S2 (Appendix A). By using the mass balance calculations between influent and AGS samples, we could calculate the SRT (θ_*x*_) and the net growth rate (μ_*x*_) of each MAG (*x*) in the AGS plant using eqs 1 and 2, respectively. The SRT was simply calculated based on dividing the biomass (measured as TSS) in the system by the biomass leaving the system. This approach assumed that the AGS plant already reached a steady state, and there was no change in the biomass (*M*_AGS_) in the AGS reactor. These calculations were conducted for both genome-resolved metagenomics and genome-resolved metatranscriptomics data sets. For metatranscriptomics mass balance calculations, we used average values of relative expressions of species (*p*_*x*,AGS_) in the anaerobic and aerobic phases.
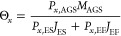
1
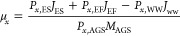
2where *p*_*x*,AGS_ is relative abundance or relative expression of MAG (*x*) in the AGS reactor (%); *M*_AGS_ is total TSS biomass in the AGS reactor = 55,972 kg. *p*_*x*,ES_ is relative abundance or relative expression of MAG (*x*) in the excess sludge (%); *J*_ES_ is total TSS biomass discharged with excess sludge per day = 2177 kg d^–1^. *p*_*x*,EF_ is relative abundance or relative expression of MAG (*x*) in the effluent (%); *J*_EF_ is total TSS biomass escaped with the effluent per day = 1256 kg d^–1^. *p*_*x*,WW_ is relative abundance or relative expression of MAG (*x*) in the influent wastewater; *J*_ww_ is daily influent TSS biomass load = 4122.5 kg d^–1^.

To calculate *P*_*x*,AGS_, we utilized the aggregate size fractionation of MLSS during the aerobic phase as it represents a well-mixed scenario. The MLSS (aerobic-well-mixed) consist of 22% LG, 53% SG, and 25% FL (Table S2; Appendix A). We applied the same mass balance model for different-sized aggregates in the AGS reactor in order to estimate the SRT (θ_*x*_) and μ_*x*_ of species separately for FL, SG, and LG, taking into consideration the sludge fraction in the excess sludge, which consisted of 50% FL, 45% SG, and 5% LG (Table S2; Appendix A).

## 3 Results

### 3.1 Ordination Plots of Metagenomics- and Metatranscriptomics-Based Community Composition Exhibit Different Proximity between Samples

A total of 681.30 million (M) (201.36 Gb) and 957.25 M (287.18 Gb) paired-end (PE) reads were obtained, respectively, for metagenomics (*n* = 18) and metatranscriptomics (*n* = 27) Illumina short read sequencing, with a minimum yield of ∼10 Gb raw sequencing data per sample (Tables S3 and S4; Appendix A). A total of 53.56 Gb raw data was generated on the Nanopore sequencing platform (*n* = 4 samples; Table S5). A total of 812 MAGs were recovered from the assembly and binning process (Appendix B). The relative abundances and expressions of these MAGs are presented in Appendix C. MAGs with abundances ≥0.1% were identified as dominant MAGs in the system; otherwise, they were defined as rare MAGs as proposed elsewhere.^6^

**Figure 1:**
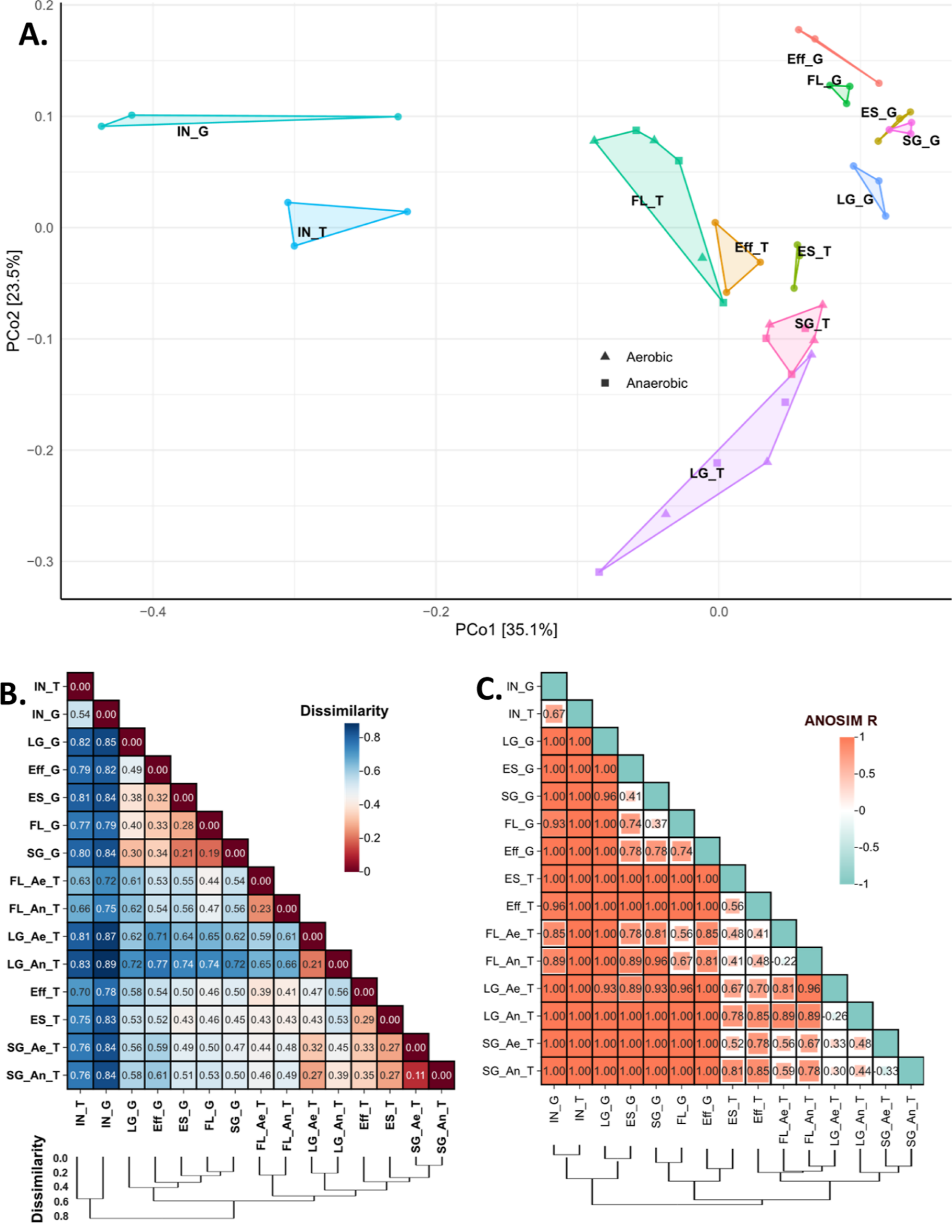
Metagenomics (G) and metatranscriptomics (T) based community composition: (A) Principal coordinates analysis (PCoA) showing similarity between influent wastewater (IN), flocs (FL), effluent (EF), excess sludge (ES), SG, and LG based on distance metric (Bray–Curtis); (B) Bray–Curtis dissimilarity distance matrix between samples (falls between 0) (identical) and 1 (completely dissimilar); (C) ANOSIM (falls between −1 and 1. A positive R value means that intergroup variation is considered significant, while a negative R-value suggests that inner-group variation is larger than intergroup variation and, therefore, no significant differences).

During the sampling period, the treatment plant exhibited stable performance with regard to COD and nutrients removal (Table S6).^39^ PCoA based on taxonomic metric (Bray–Curtis) using both metagenomics and metatranscriptomics data sets (Figure 1A) revealed differences in community composition (i.e., beta-diversity) between the different-sized aggregates and between influent and different aggregates. Overall, PCoA plots of metagenomics and metatranscriptomics exhibited the same general layout of samples (Figure 1A) but different proximity among samples (Figure 1B). The floc samples were in closer proximity to the influent samples, followed by small and large granule samples. This observation indicates that FL samples exhibited the greatest similarity to influent samples followed by SG and LG, or in other words, the immigration from the source community was likely higher to flocculent sludge compared to that from granular sludge. However, the metatranscriptomics data show that the samples are much closer to each other, compared to the metagenomics data where the influent samples were positioned distant from the FL, SG, LG, excess sludge, and effluent samples (Figure 1A). For example, the dissimilarity distances between influent and FL, SG, and LG were 0.79, 0.84, and 0.85, respectively, for the metagenomics data, compared to 0.63, 0.76, and 0.81, respectively, for the metatranscriptomics data (Figure 1B). This indicates that immigration had a stronger impact on the shaping of microbial community assembly in the case of metatranscriptomics-based community composition. The SG samples showed more proximity to the LG than the FL samples for metatranscriptomics, as opposed to metagenomics where SG were closer to the FL samples. This indicates that FL and SG were more similar in terms of metagenomics-based community composition, while SG and LG were more similar in terms of metatranscriptomics-based community composition when compared to metagenomics analysis. As expected, excess sludge samples were positioned in the middle of flocs and SG samples in both PCoA plots because it comprised mainly flocs (50%) and SG (45%) with minor contribution from LG (5%) (Table S2: Appendix A). The effluent samples had much higher similarity with the FL samples, as shown in both PCoA plots. This was due to FL biomass escaping easier in the effluent on the top of the reactor during the feeding phase of the SBR operation. In general, metagenomics and metatranscriptomics samples clustered in different zones and showed distinct differences. Furthermore, there was a clustering based on the sample type, as shown in the PCoA plot. However, the variability in metatranscriptomics samples was higher than that in metagenomics samples, reflecting the influence of gene expression variability under different conditions. There were high similarities observed in the community composition of metatranscriptomics samples between aerobic and anaerobic conditions within the same aggregate size, as indicated by PCoA plot and supported by both dissimilarity measures and ANOSIM analysis (Figure 1B and C).

### 3.2 Assessing the Growth Rate of Species in AGS System

**Figure 2:**
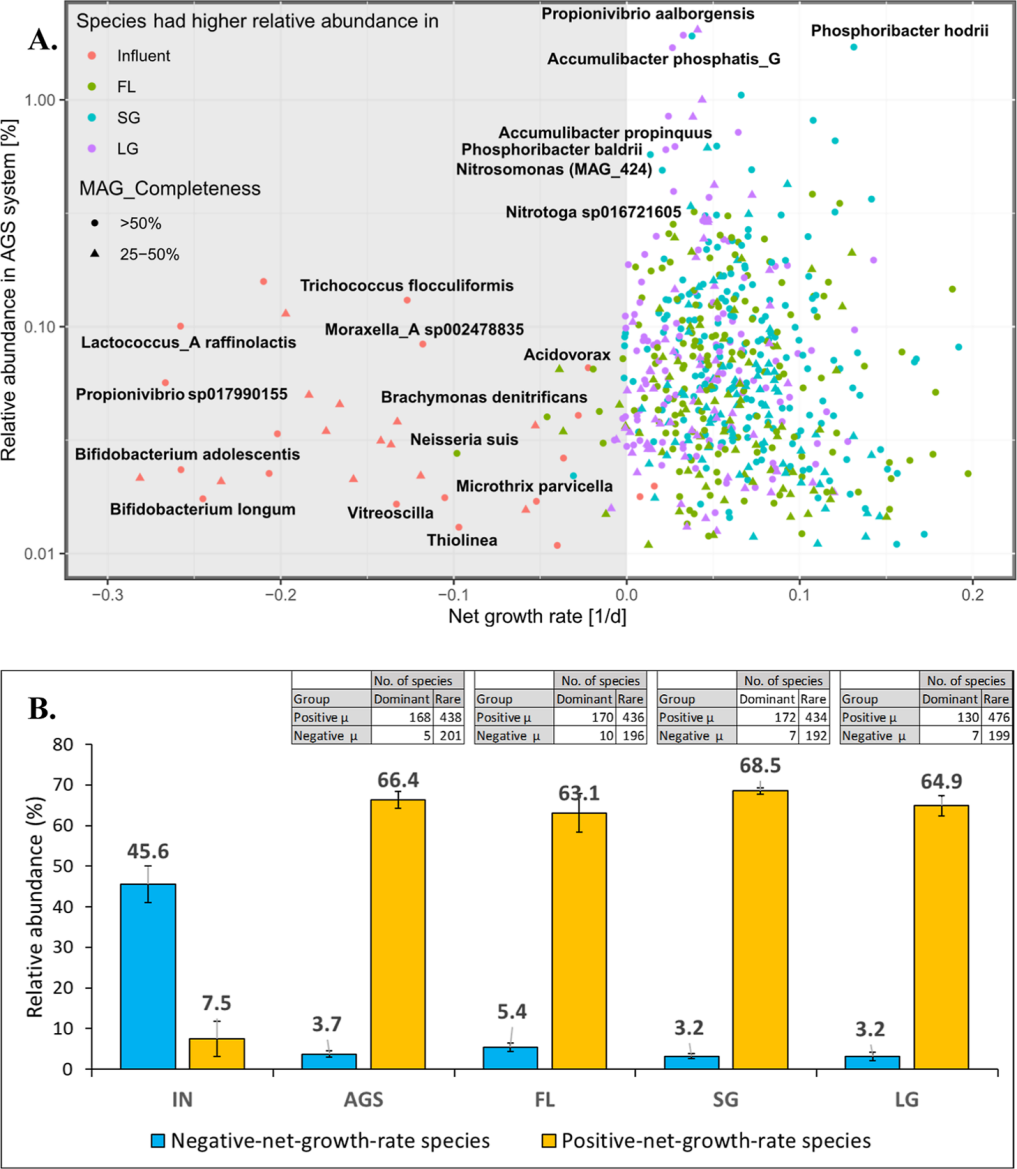
Positive- and negative-net-growth-rate species in the AGS system based on metagenomics mass balance (*n* = 3). (A) Net growth rate vs relative abundance (%) of species in AGS samples (25% FL, 53% SG, 22% LG). Species with growth rates < −0.3 d^–1^ (*n* = 152) were excluded for plot clarity. All labeled species have completeness > 50% and contamination < 10% as per minimum information about a MAG (MIMAG).^38^ (B) Relative contribution of immigration, shown as cumulative relative abundance in influent (IN) and AGS samples, and across aggregate sizes (FL, SG, LG). Mapped reads account for an average of approximately 70%. MAGs with completeness >50% (*n* = 540) represent an average of 75% of the mapped reads across all samples.

The immigrants in the current study are defined as the bacteria shared between the influent and the AGS system. Species with negative net-growth rates decline in the AGS system and persist only through passive transport and immigration. The relative contribution of immigration in community assembly of the AGS system was studied and quantified through metagenomics-based and metatranscriptomics-based mass balance approaches (calculation sheets: Appendix D). The metagenomics-based mass balance identified that about 206 ± 19 species (5 dominant and 201 rare) had negative net-growth rates (or decay rate; <0 d^–1^) (Figure 2A and Appendix D). These species had a lower cumulative relative abundance in the AGS system (3.7 ± 0.76%) while having a higher cumulative relative abundance in the influent (45.6 ± 4.46%) (Figure 2B). The total daily biomass load (measured as TSS) transported from the influent load into the AGS system was estimated to be about 7.5% of the total AGS biomass (Appendix D). The reason these organisms are constantly detected in the system is due to their continuous immigration via the influent.

On the other hand, there were more species with positive net-growth rates (*n* = 606 ± 19, 168 dominant, 438 rare; Figure 2A), which had higher cumulative relative abundances in the AGS system (66.4 ± 2.1%) but relatively lower cumulative abundance in the influent (7.5 ± 4.4%) (Figure 2B). The majority of negative-net-growth-rate species in the AGS system had higher abundance in the influent samples than in the AGS samples (FL, SG, and LG), while a few of the positive-net-growth-rate species in the AGS system had higher relative abundance (>0.1%) in the influent samples (Figures 2A, S2).

**Figure 3:**
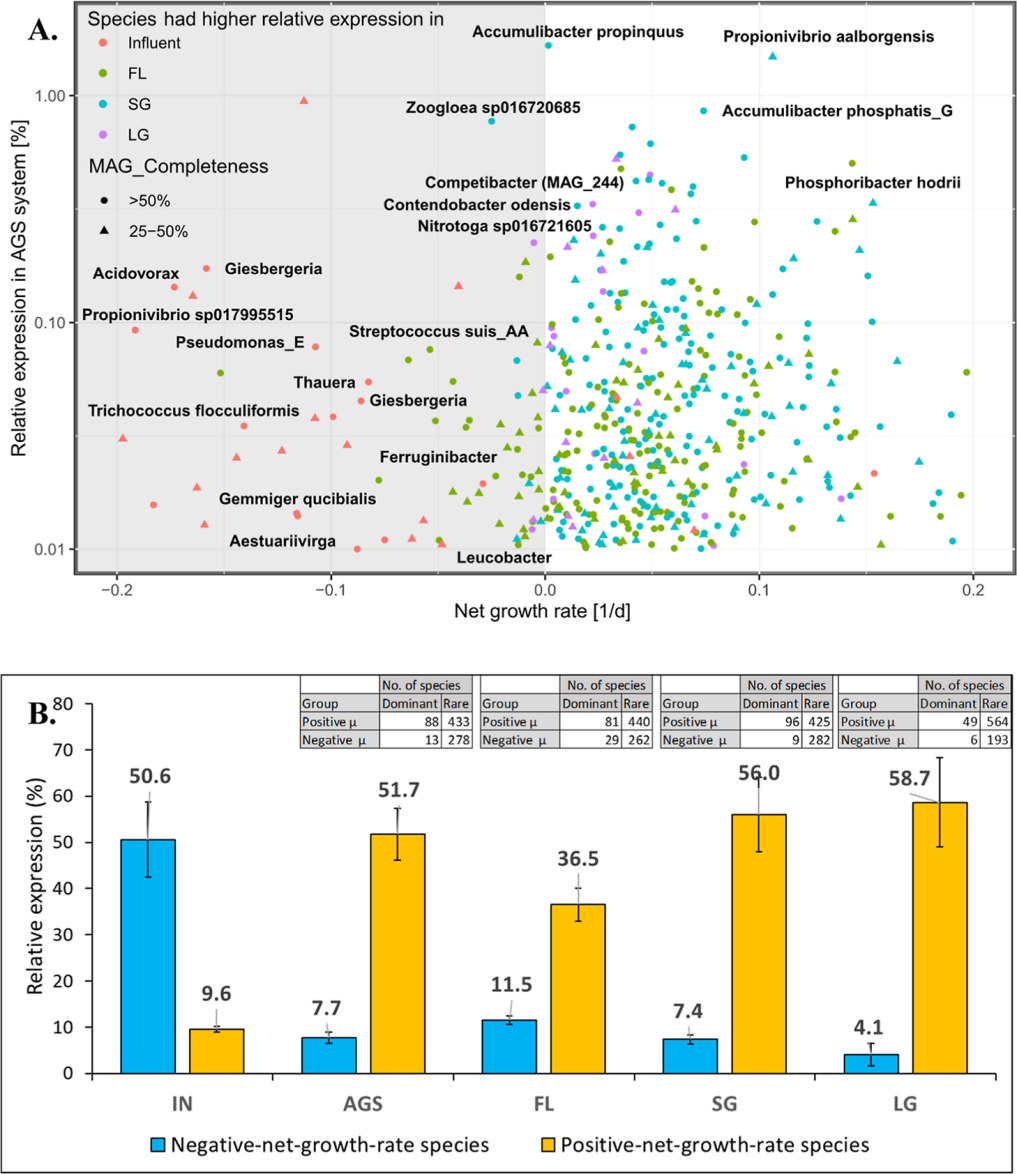
Positive- and negative-net-growth-rate species in the AGS system based on metatranscriptomics mass balance (*n* = 3). (A) Net growth rate vs relative expression (%) of species in AGS samples (25% FL, 53% SG, 22% LG). Species with growth rates < −0.2 d^–1^ (*n* = 221) were excluded for plot clarity. All labeled species have completeness >50% and contamination <10% as per minimum information about a MAG (MIMAG).^38^ (B) Relative contribution of immigration, shown as cumulative relative expression in influent (IN) and AGS samples, and across aggregate sizes (FL, SG, LG). Mapped reads account for an average of approximately 60%. MAGs with completeness >50% (*n* = 540) represent an average of 75% of the mapped reads across all samples.

The metatranscriptomics-based mass balance exhibited a higher immigration rate than metagenomics-based mass balance, as the number of negative-net-growth-rate species increased to 291 ± 43 (13 dominant, 278 rare) (Figure 3A). This is in agreement with the metatranscriptomics-based PCoA plot, which shows the influent samples (source of immigrants) were closer to AGS samples (FL, SG, LG) as compared to metagenomic-based PCoA plot (Figure 1A). Similarly, these species had low corresponding cumulative relative expression in the AGS system (7.7 ± 1.2%), compared to the influent (50.6 ± 8.1%) (Figure 3B). Then again, there were more positive-net-growth-rate species in the AGS system (n = 521 ± 43, 88 dominant, 433 rare; Figure 3A), which had higher cumulative relative expression in the AGS system (51.7 ± 5.6%), compared to the influent (9.6 ± 0.62%) (Figure 3B). Likewise, using the metagenomics-based mass balance, the majority of negative-net-growth-rate species in AGS system had higher relative expression in the influent samples than the AGS samples (FL, SG, and LG) (Figures 3A, S3). There were also a few positive-net-growth-rate species in the AGS system that had higher relative expression (>0.1%) in the influent (FL, SG, and LG) (Figures 3A, S3).

The impact of immigration was then quantified individually for the differently sized microbial aggregates. All negative-net-growth-rate species in the different aggregate sizes (FL, SG, and LG) had lower cumulative relative abundances and expressions in the AGS system, compared to higher values in the influent (Figures 2B, 3B). In contrast, positive-net-growth-rate species had higher cumulative abundances and expressions in the AGS system and lower values in the influent (Figures 2B, 3B). Both methods revealed that the cumulative relative abundances and expressions for negative-net-growth-rate species were higher in the FL followed by the SG and then the LG, indicating that the immigration from the source community was higher to flocculent sludge compared to granular sludge (Figures 2B, 3B). However, the differences in the immigration rate between flocculent and granular sludge was only statistically significant (*P* < 0.05) in the case of the metatranscriptomics mass balance method where the immigration rate was 11.5 ± 0.9% for FL versus 7.4 ± 1 and 4.1 ± 2.4% for SG and LG, respectively.

**Figure 4:**
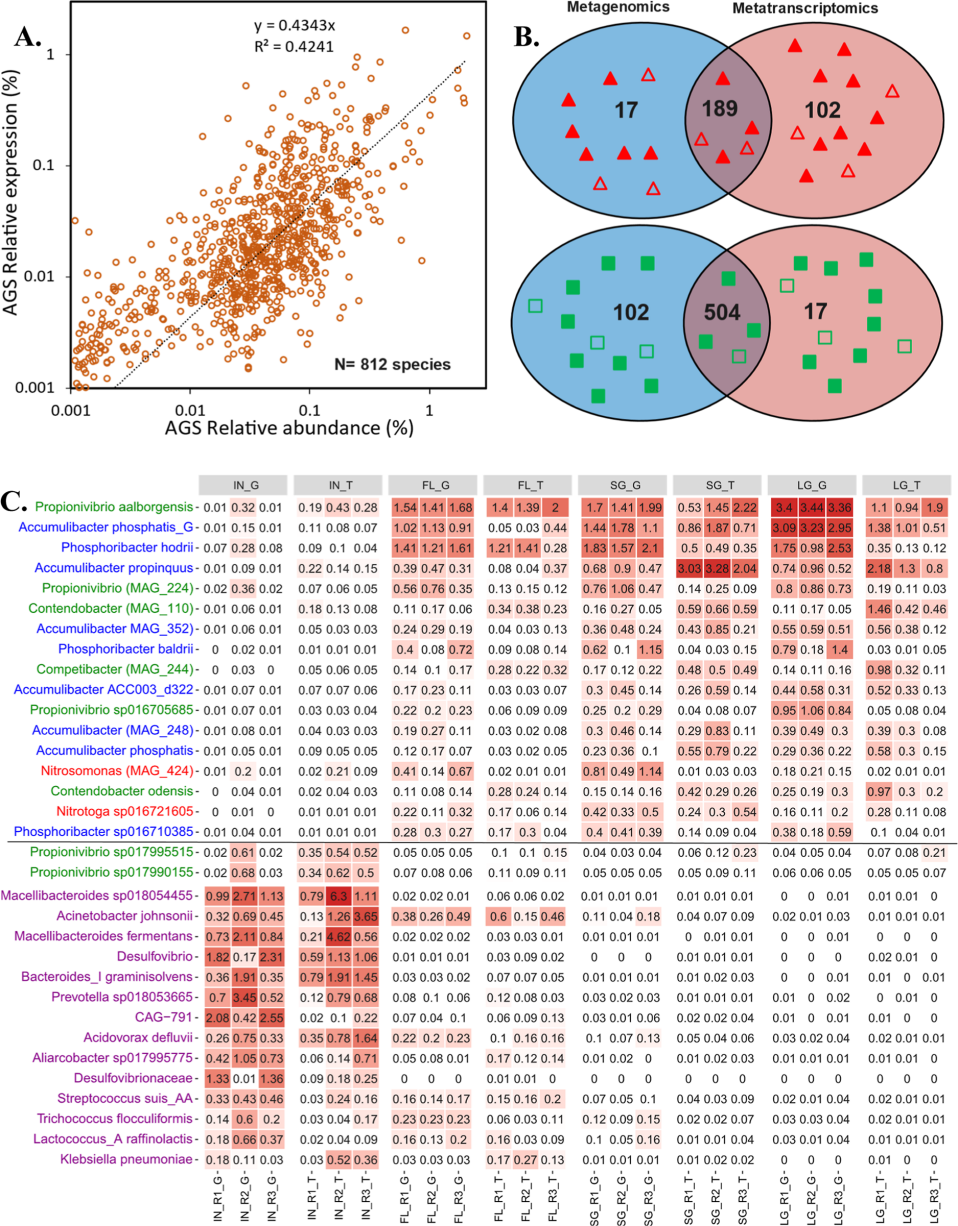
(A) Correlation of relative abundance and relative expression of species in AGS system (25% FL, 53% SG, 22% LG). (B) Number of negative- (▲) and positive- (■) net-growth-rate species shared between metagenomics and metatranscriptomics mass balance calculations. (C) Heatmap distribution [abundances (G) and expressions (T)] of key functional groups (PAOs: blue, GAOs: green, nitrifiers: red, fecal- and sewage infrastructure-derived microbes: purple) in influent, flocs, SG, and LG. All listed MAGs have completeness >50% and contamination <10% according to minimum information about a MAG (MIMAG).^38^

There was a positive linear relationship between relative abundances and relative expressions of species in the AGS system (Figure 4A). However, the two parameters are not strongly correlated (*R*^2^ = 0.42), especially at low values. Therefore, the two methods yielded different immigration rates. The number of positive- and negative-net-growth-rate species in the AGS system shared between the two methods was 504 and 189, respectively (Figure 4B). Most of the positive-net-growth-rate species in the AGS system identified across the two methods belonged to the phylum Pseudomonadota (previously known as Proteobacteria) (*n* = 175, 35%), followed by Bacteroidota (*n* = 144, 29%) and Actinobacteriota (*n* = 36, 7%) (Figure S4). Many important functional groups responsible for carbon, nitrogen, and phosphorus removal belong to the phylum Pseudomonadota such as polyphosphate-accumulating organisms (PAOs) (i.e., *Ca. Accumulibacter*), glycogen-accumulating organisms (GAOs) (i.e., *Propionivibrio*, *Ca. Competibacter*, and *Ca. Contendobacter*). and nitrifiers (i.e., *Nitrosomonas* and *Nitrotoga*). Actinobacteriota also include important functional genera such as *Tetrasphaera* and *Ca. Phosphoribacter* (previously identified as *Tetrasphaera*) whose members are considered as PAOs.^40^ With the exception of a few cases, most of these functional groups had positive net-growth rates in the AGS system according to the two approaches (Appendix E) in which their growth was primarily driven by deterministic factors in the AGS system. These species had very low abundances and expressions in wastewater while were progressively enriched and expressed with an increase in microbial aggregates size (Figure 4C).

On the other hand, most of the negative-net-growth-rate species identified across the two methods (189 species) belonged to the phyla Bacillota (previously known as Firmicutes) (*n* = 71, 38%), Bacteroidota (*n* = 33, 18%), Pseudomonadota (*n* = 24, 13%), and Desulfobacterota (*n* = 11, 6%) (Figure S4). Most of these species are considered as fecal- and sewage infrastructure-derived microbes (i.e., originating from human gut/gastrointestinal tract and sewers due to anaerobic environment).^6,41^ These include fermentation bacteria (i.e., species of the genera *Macellibacteroides*, *Paludibacter*, *Prevotella*, *Proteocatella*, *Streptococcus*, *Aliarcobacter*, *Cloacibacterium*, and *Bacteroides*) and sulfate reducing organisms (*Desulfobacter* and *Acidovorax*) (Appendix E, Figure 4C). Also, negative-net-growth-rate species involved 3 species belonging to fermentative GAOs [*Propionivibrio* sp1799155, *Propionivibrio* sp*17995515*, and *Propionivibrio* (MAG_723)] (Appendix E, Figure 4C). These species exhibited higher relative abundances and expressions in the influent, which progressively diminished in AGS as the microbial aggregate size increased, with flocs showing greater similarity to the influent (Figure 4C). These species could only persist in the AGS system due to passive transport and immigration. Notably, species with positive net-growth rates did not include any phyla belonging to Bacillota or Desulfobacterota (Figure S4), indicating a decline of these phyla in the AGS system.

### 3.3 Assessing the Metabolic Activities of Immigrants

**Figure 5:**
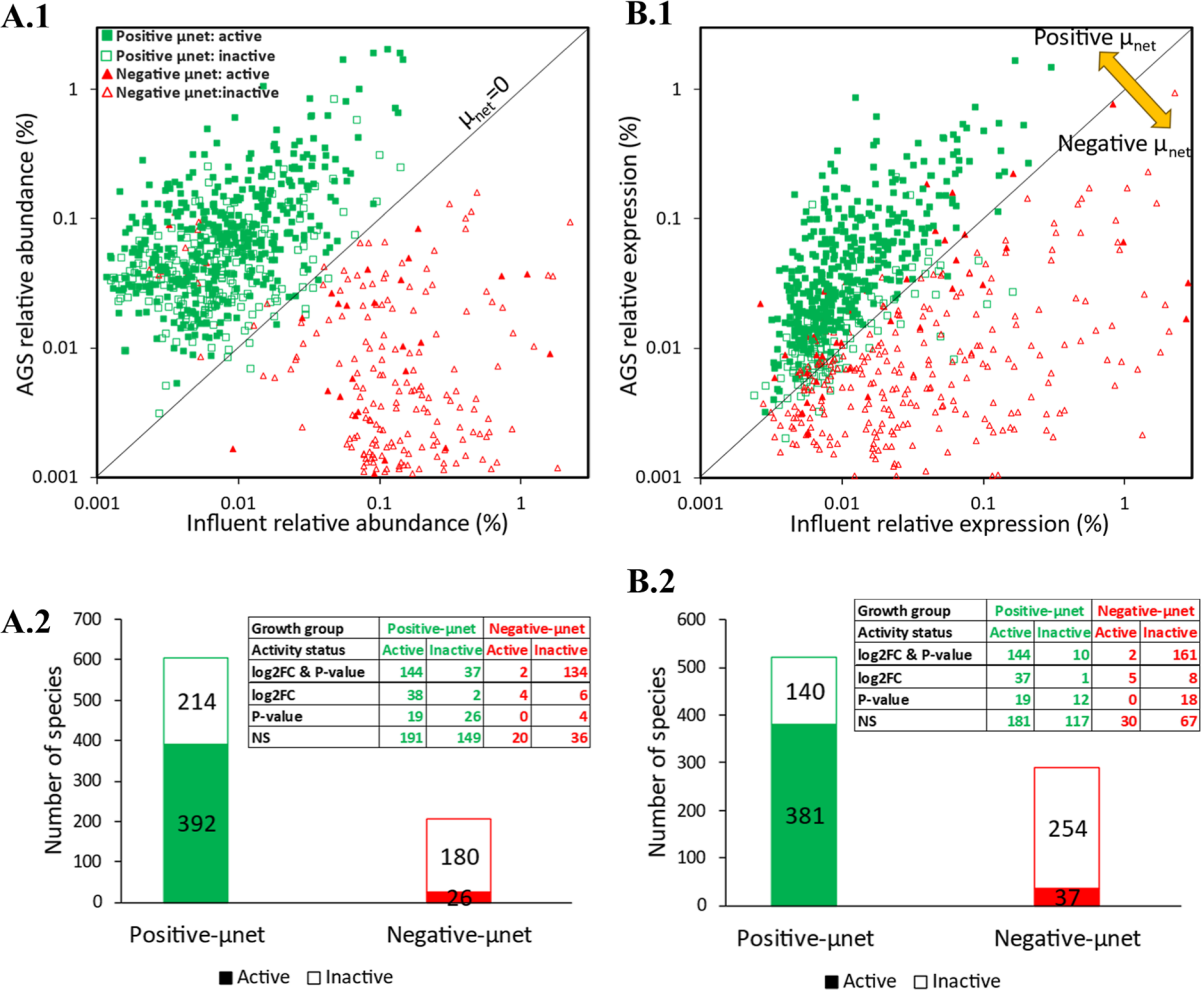
Classification of positive (green square) and negative (red triangle) net-growth-rate species in AGS system derived through metagenomics (A) and metatranscriptomics (B) mass balance models into active (filled shape) and inactive members (empty shape). (A.1) Correlation of relative abundance in influent and AGS system. (A.2) Corresponding number of active and inactive species for each group from plot (A.1). (B.1) Correlation of relative expression in influent and AGS system. (B.2) Corresponding number of active and inactive species for each group from plot (A.2). The groups were further classified into significant and nonsignificant (NS). Significant species indicates differences between influent and AGS samples had absolute log_2_ fold change (log_2_ FC) ≥ 1 and/or *P*-value < 5%, otherwise were defined as NS.

The net-growth rate parameter alone is insufficient to fully determine the microbial growth or activity of immigrants. For example, negative-net-growth-rate species can be very active and responsible for all of the activity in a reactor given certain extreme conditions. The metabolic activities of these species were examined by carrying out direct comparisons in MAGs’ expressions between influent and the AGS compartments using DESeq2 software. The results revealed that most species with a negative net-growth rate were inactive, with only 26 out of 206 species and 37 out of 291 species exhibiting higher activity as identified through metagenomics and metatranscriptomics mass balance models, respectively (Figure 5). Among these species were *Acetomicrobium* sp*012518015*, *Novosphingobium* sp*020853995*, *Flavipsychrobacter*, *Aureliella*, and *Lacunisphaera*, *Acinetobacter*, *Lacunisphaera*, *Zoogloea* sp*016720685*, *Draconibacterium*, *Flavobacterium*, *Ferruginibacter*, *Aquirickettsiella isopodorum,* and *Microbacterium* (Appendix E). However, only 6–7 species showed significant differences (|log_2_ FC| ≥ 1 and/or *P*-value < 5%; Figure 5A.2 and B.2).

On the other hand, species with a positive net-growth rate comprised a larger proportion of active members (392/606 for metagenomics, 381/521 for metatranscriptomics), highlighting their significant contribution to the activities and performance of the AGS system (Figure 5). Interestingly, the metatranscriptomics mass balance model identified a higher proportion of active members and effectively categorized more inactive members into the negative-net-growth-rate group (Figure 5B.2). This is likely due to the model’s reliance on relative expression levels in its calculations. Therefore, the metatranscriptomics mass balance approach proves to be a valid method for determining both growth classification (positive vs negative) and activity status (active vs inactive).

It is important to note that our DESeq2 analysis was conducted by using metatranscriptomics data without normalization against metagenomics data. One could argue that positive-net-growth species exhibit higher expression (activity) in the AGS system due to their higher relative abundances and vice versa for negative-net-growth species. For a specific species, the relative expression (RNA) reflects its overall activity, while the relative abundance (DNA) represents its population size. Therefore, the RNA/DNA ratio serves as a normalized measure of species activity per unit, referred to as “relative activity.” Using this approach, we observed that a larger fraction of species in the negative-net-growth-rate group had higher relative activity (83% and 65% for species identified through metagenomics and metatranscriptomics mass balance models, respectively) compared to the positive-net-growth-rate group (49% and 53% for species identified through metagenomics and metatranscriptomics mass balance models, respectively) (Figures S5C2, S6C2). Since the mapped reads in the metatranscriptomics data were fewer than in the metagenomics data, we used an RNA/DNA cutoff value of 0.434 (calculated as the average across species and derived from the slope of Figure 4A) to delineate species with higher and lower relative activity. While using an RNA/DNA cutoff value of 1 would reduce the number of species classified as having higher relative activity in both positive- and negative-net-growth groups, the negative-net-growth group would still maintain a higher fraction of species with elevated relative activity compared to the positive-net-growth group (Figures S5B, S6B). This strongly indicates that negative-net-growth species, which persist due to immigration, significantly contribute to AGS activity.

**Figure 6:**
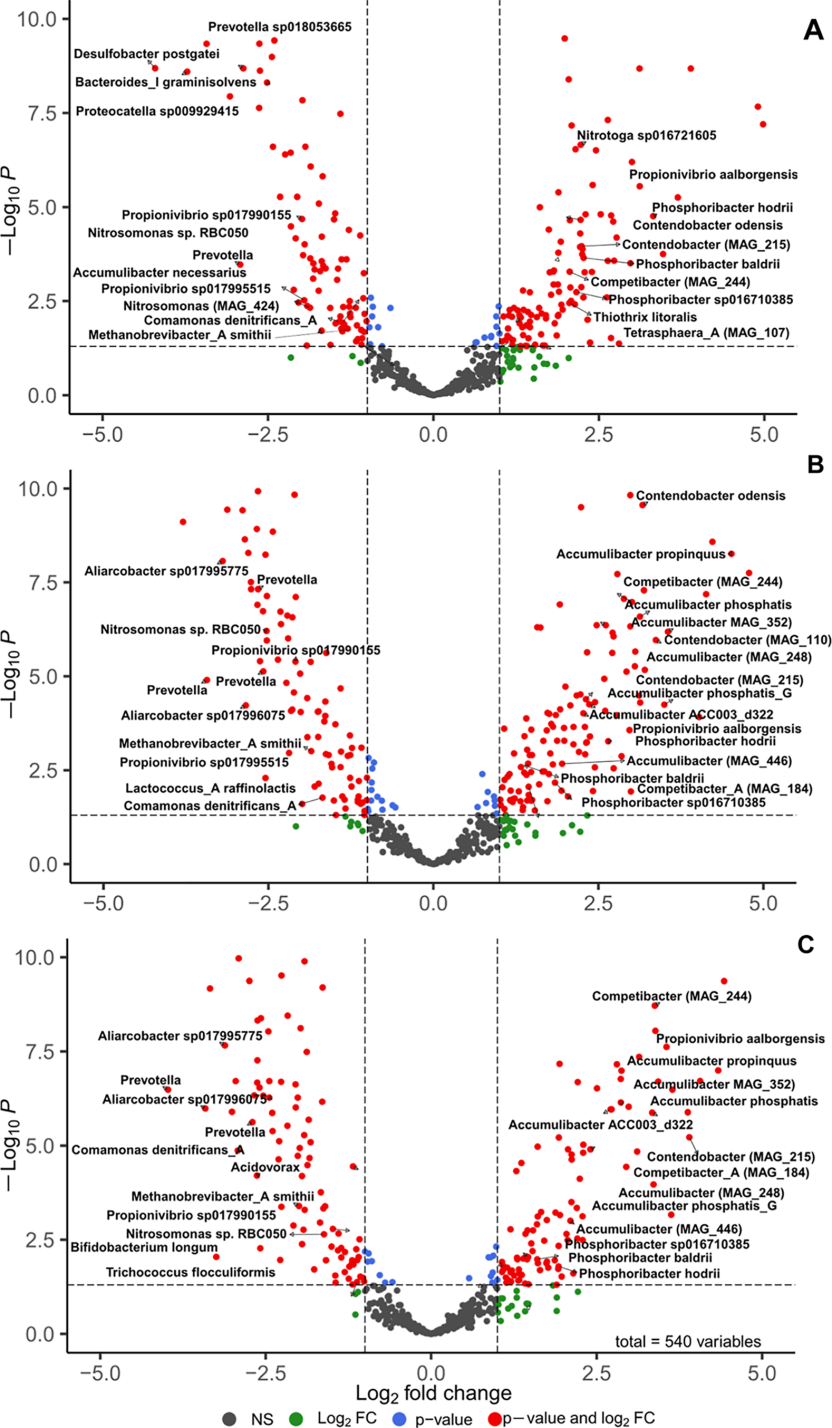
EnhancedVolcano plot showing the results of differential expression analyses between influent (IN) and different-sized microbial aggregates in the AGS system: (A) Flocs (FL); (B) SG; and (C) LG. Species are colored based on the level of significance and degrees of variation; either as absolute log_2_ fold change |log_2_ FC| ≥ 1 or *P*-value < 5% or meeting both |log_2_ FC| ≥ 1 and *P*-value <5% or meeting none of the previous conditions as nonsignificant (NS). Species with negative log_2_ FC indicate they were downregulated (inactive) in the AGS plant or active in the influent. While positive log_2_ FC values indicate they were upregulated (active) in the AGS system. Only MAGs with completeness >50% and contamination <10% (*n* = 540 species) are displayed as per minimum information about a MAG (MIMAG).^38^

Differential expression analysis using DESeq2 was also performed to compare the microbial activity between influent and different-sized microbial aggregates (FL, SG, and LG). Our analysis revealed that some important functional species which are typically found in wastewater treatment systems had higher activity in the influent as compared to the different-sized microbial aggregates in the system (Figure 6). These species are considered immigrants, constantly seeding the AGS bioreactor with important functional groups. These included GAOs (*Propionivibrio* sp1799155 and *Propionivibrio* sp*17995515*), ammonium oxidizing bacteria (AOB, *Nitrosomonas* sp. *RBC050*), fermentation/anaerobic bacteria (*Bacteroides*, *Proteocatella*, *Prevotella*, *Aliarcobacter*), anaerobic archaea (*Methanobrevibacter*), and sulfate reducing bacteria (*Desulfobacter* and *Acidovorax*) (Figure 6). Fermentation, anaerobic, and sulfate-reducing organisms were expected to have higher activity in the influent because of the anaerobic conditions such as sewers environment. Some GAOs species may have higher activity in the influent because of the high volatile fatty acid (VFA) generated from the fermentation process or because these GAOs themselves performed the fermentation process.^42,43^ In the gravity sewer biofilm, there can be aerobic/anaerobic cycling due to different hydraulic flows (water levels) during the day, which can be a reason for the growth/activity of GAOs and PAOs in biofilms on the sewer walls, resulting in their presence in the influent. Furthermore, one species of AOB (*Nitrosomonas* sp. *RBC050*) appeared to have higher activity in the influent compared to the FL, SG, and LG, although it had positive-net-growth rate in the AGS system (Figure 6B & C). It is not fully understood why this species had a higher activity in the influent. Higher AOB activity in the influent indicates that there may be (temporarily) aerated niches in the biofilm grown on the gravity sewer, e.g., due to variable water depth. In contrast to GAOs, no PAO species exhibited higher activity in the influent compared to the FL, SG, and LG samples, except for *Accumulibacter necessarius* (Figure 6A). This species showed higher activity in the influent than in the FL samples, despite having a positive net-growth rate. All other PAOs demonstrated positive growth and activity within the AGS reactor, driven by deterministic factors such as the cyclic anaerobic–aerobic regime rather than immigration.

Overall, compared to the SG and LG samples, the FL samples contained fewer functional groups with higher activity than the influent. For instance, many PAOs, including species from the genera *Ca. Accumulibacter*, *Ca. Phosphoribacter*, and *Tetrasphaera*, showed higher activity in the SG and LG samples than in the influent (Figure 6B & C). In contrast, FL samples contained only a few species from fermentative PAO genera, such as *Tetrasphaera* and *Ca. Phosphoribacter*, which displayed higher activity in the FL than in the influent (Figure 6A). This suggests that environmental conditions shaping PAOs growth had a more substantial influence on granular sludge, while flocculent sludge was more affected by immigration.

## 4 Discussion

Environmental microbiologists are consistently intrigued by the relative influence that immigration and species sorting have on structuring the microbial community assembly in an ecosystem. Previous research has attempted to investigate the influence of immigrant populations on shaping microbial communities in various natural ecosystems,^11,44^ including lakes, rivers, and glaciers, and engineered ecosystems such as wastewater treatment plants.^6,45−50^ Understanding this influence in engineered wastewater treatment systems is crucial, as incoming immigrants might significantly affect the functionality of the treatment system. The cost of next-generation sequencing technologies rapidly decreases, which enables us to use more advance techniques such as metagenomics and metatranscriptomics to significantly improve our understanding and functions of various engineered ecosystems, which can help engineers to better design and operate biological WWTPs.^51^

### 4.1 Different Approaches Revealed Different Immigration Rates

In this study, genome-resolved metagenomics and metatranscriptomics techniques were employed to comprehensively evaluate the contribution of the immigrant population in structuring the microbial community assemblages of different-sized microbial aggregates in a full-scale AGS system. The metatranscriptomics-based mass balance exhibited a higher immigration rate (7.7 ± 1.2%) than metagenomics-based mass balance (3.7 ± 0.76%). These values could increase to 13% and 5%, respectively, if the percentage of unmapped reads (30–40%) is considered. This result aligns with previous studies, which reported that approximately 10% of taxa were primarily present due to immigration.^12^ The metagenomics- and metatranscriptomics-based community compositions provided different perspectives about the level of immigrant population probably due to the variation of RNA/DNA ratios (Figure 4A). These discrepancies may arise from transcriptional regulation differences between microbial species, which often result in variations in relative expression that are independent of relative abundance. Environmental factors, such as substrate availability (e.g., access to slowly vs readily degradable substrate), oxygen levels, and nutrient gradients, significantly influence gene expression levels and may cause differences in activities, creating unique metabolic niches within microbial aggregates. In our study, we used genome-level relative expression (aggregated expression of all genes within a MAG), which is far less susceptible to rapid temporal fluctuations compared to individual gene-level expression. As shown in DESeq2 comparisons (Figure S7), variability at the genome level remains stable between aerobic and anaerobic phases (Figure S7A), unlike gene-level expression that can change over short time scales (Figure S7B). This justifies our focus on genome-level data to evaluate the metabolic activity and viability of species. Previous studies also revealed that different sequencing/metaomics methods can provide different views about the microbial community composition. For example, Kleikamp et al.^52^ studied the microbial composition of AGS for 3 different WWTPs using metagenomics, metaproteomics, and 16S rRNA sequencing. Their study found that the relative abundances of bacteria varied significantly between methods, especially at lower taxonomic levels. It is worth noting that the mass balance calculations in this study were based on TSS measurements. The use of DNA and RNA yields or real cell concentration values from the influent, effluent, and excess sludge can better estimate the relative contribution of immigration. Nevertheless, the relative comparison between the two methods can still be valid.

### 4.2 Immigration from the Influent Community was Higher to Flocculent than Granular Sludge

The metagenomics-based mass balance approach revealed a greater impact of immigration in shaping the microbial community of flocculent sludge (FL; 5.4 ± 1.1%) compared to granular sludge (SG; 3.2 ± 0.6% and LG; 3.2 ± 1%) (Figure 2B). The metatranscriptomics-based mass balance approach showcased a consistent trend in immigration with slight variations, demonstrating a greater influence in shaping the microbial community within flocculent sludge (11.5 ± 0.9%) compared to granular sludge (SG: 7.4 ± 1% and LG: 4.1 ± 2.4%) (Figure 3B). This is justified by the fact that the flocculent communities depicted a greater resemblance to the influent communities in comparison to granular communities (Figure 1). This probably occurs because the incoming community is easily incorporated in the floc fraction, then enmeshed to some extent in the granular sludge fraction. Nevertheless, these incoming communities to the floc fraction are probably decaying and not establishing themselves in the process. The effect of immigration being lower in granular sludge is also possibly due to the fact that granular sludge (SG and LG) has longer SRTs than flocculent sludge (FL). The metagenomics-based mass balance approach revealed different SRTs for different-sized microbial aggregates (FL; 12 ± 5 days, SG; 24 ± 7 days, and LG; 60 ± 9 days) (Appendix D). In metacommunity ecology, systems characterized by short retention times, typically contain a microbial community structure that is notably impacted by immigration, a phenomenon observed in inland natural water ecosystems such as streams, estuaries, and lakes.^53^ However, wastewater treatment systems are engineered to have a longer SRT (by separating it from the hydraulic retention time) by the systematic retention of microbial aggregates within the process. Vuono et al.^49^ reported that microbial communities in sludge with short SRTs are more influenced by immigration as compared to those in long SRTs in a full-scale CAS system where the SRT was progressively decreased from 30 to 3 days. Vuono et al.^49^ further noted that the immigration trend reversed when the SRT was extended from 3 to 30 days due to the increased influence of species sorting metacommunity paradigm as opposed to immigration. At long SRT there is more decay time for the incoming community and more time for positive-net-growth species to form biomass. Therefore, the ratio of negative decaying immigrants to a positive growing indigenous community decreases with increased SRT.

### 4.3 Environmental and Operational Conditions of AGS Determine the Growth and Decline of Immigrants

In this study, all species detected in AGS were also present in the influent, albeit at very low abundances. However, relying solely on the concept of shared taxa between the influent and CAS or AGS is insufficient to accurately describe and quantify the relative contribution of immigration, as highlighted in previous studies.^12,54^ Gibson et al.^14^ introduced the concept of adding a sterile substrate (i.e., without microbial communities in the influent) as a control in their study to accurately assess the impact of immigration. Their study revealed that the majority of immigrants in CAS exhibited low or negative net-growth rates, accounted for 4–14% of the reads, and persisted primarily due to mass immigration. These findings align with our results, which revealed that species with negative net-growth rates accounted for 5% and 13% of the mapped reads in the metagenomics and metatranscriptomics data, respectively. On the other hand, the study of Gibson et al.^14^ introduced the term “resident genera” to describe species that persist over time without the need for continuous seeding or immigration. These species exhibited positive net-growth rates and accounted for 75% and 77% of the reads. This suggests that the enrichment of positive-net-growth species in our study is not attributed to immigration but is primarily driven by environmental conditions and deterministic selection factors, referred as “species sorting”. In particular, heterotrophic mass-flow immigrants appear to be heavily influenced by deterministic factors such as substrate availability and operational parameters.^14,55^ In addition, alternating anaerobic feast and famine strategy and long SRT operation promote the growth of slow-growing organisms in AGS such as nitrifiers, PAOs, and GAOs.^56,57^ These species exhibited very low abundances and expression levels in the influent but were progressively enriched and expressed as the microbial aggregate size increased (Figure 4C). On the other hand, the decline of gut microbes, fermentation, and sulfate-reducing bacteria in the AGS system (Figure 4C) may be attributed to differences in niche availability between the influent wastewater and the AGS system. These species thrive in the anaerobic sewer environment but may have limited tolerance to the aerobic conditions of AGS.

### 4.4 Negative-Net-Growth-Rate Populations Could be Very Active and Growing Populations May Still be Impacted by Immigration

Differential expression analysis using the DESeq2 approach, which directly compared species expression between the influent and AGS systems, revealed that most positive-net-growth species were upregulated (active) in the AGS system. A high proportion of negative-net-growth species were downregulated (inactive) in the AGS system compared with their expression in the influent. However, a smaller subset of species exhibited high activity, underscoring the importance of immigration in contributing to the AGS system activity. For instance, certain species within the genera *Acinetobacter* (associated with biological phosphorus removal),^58^*Zoogloea* and *Ferruginibacter* (linked to floc formation and EPS production),^59^*Novosphingobium* (known for recalcitrant pollutant degradation),^60^*Flavobacterium* (involved in organic matter degradation), and *Acetomicrobium* (related to anaerobic digestion and acetate production) demonstrated increased activity despite having negative net-growth rates.

Our study also revealed that negative-net-growth-rate species exhibited a higher fraction of species with elevated relative activity (RNA/DNA) compared with positive-net-growth-rate species. Previous studies on immigration, which predominantly relied on amplicon sequencing and mass balance models, concluded that this fraction of the immigrant community was inactive and did not contribute to the metabolism of activated sludge.^6,12,15^ However, our findings indicate that negative-net-growth-rate species were actively growing, even though their overall abundance declined in the AGS system. Overall, negative-net-growth-rate species had a relative activity of 2.1 (7.7/3.2), which is approximately 2.5 to 3 times higher than that of positive-net-growth-rate species (0.78, calculated as 51.7/66.4). This, for instance, suggests that negative-net-growth-rate species could putatively achieve higher substrate conversion rates per unit biomass in the AGS system compared to positive-net-growth-rate species. Guo et al.^61^ showed that the high immigration organisms are more likely to have high RNA and high polyhydroxyalkanoate (PHA) levels, which appears to be in line with the high activity of negative-net-growth-rate species of the current study. Guo et al.^61^ suggested that these organisms could be better at consuming readily degradable substrates. Our results are also consistent with a recent study that employed a sterile substrate control to experimentally distinguish active and inactive portions of the immigrating community.^14^ That study empirically demonstrated that despite displaying a negative net-growth rate active immigration-dependent genera were growing and consuming substrates within the activated sludge, as their abundance was higher in reactors receiving regular influent solids compared to those receiving sterilized influent solids. Guo et al.^62^ estimated that these high immigration populations (mostly negative growth rates) could consume up to 15% of the influent resources depending on the assumptions.

The use of relative activity (RNA/DNA) in this study comes with certain challenges and limitations. For instance, there is noticeable data noise in regions where relative abundances and expressions are low (<0.01; Figure 4A). This region, predominantly representing negative-net-growth-rate species, tends to have higher RNA/DNA values, which could introduce bias in the comparison. Additionally, when relative expression/abundance is used instead of absolute expression/abundance to calculate the ratio, it becomes critical to judge whether a certain species is genuinely active or inactive, as DNA and RNA yields can vary between samples. Instead, we can compare the relative activity of one species to that of other species. Furthermore, there is no universal RNA/DNA threshold value to categorically define active versus inactive species, as this threshold may vary across different species. This variability underscores the need for context-specific evaluations when interpreting RNA/DNA ratios.

Overall, negative-net-growth-rate species exhibited a cumulative relative expression of 7.7 ± 1.2% (rising to 13% when considering unmapped reads), highlighting their significant contribution to the activity of the AGS system. Future studies on immigration should focus on identifying which genes are expressed within each growth group to validate whether negative-net-growth-rate species play a functional role in the AGS system. It is also possible that these species were under stress, expressing genes that may not directly benefit their growth or the functionality of the AGS system such as aging or cell death-related genes. Understanding the specific gene expression patterns of these species will provide deeper insights into their roles and contributions to the AGS dynamics.

### 4.5 Activity Status of Important Functional Groups in Influent and AGS System

Our results highlighted higher activity among specific GAOs in the influent such as fermentative GAOs like *Propionivibrio* (*Propionivibrio* sp1799155 and *Propionivibrio* sp*17995515*). These were identified as negative-net-growth species in the AGS system and were relatively more active in the influent. The higher COD/P ratio (i.e., >100 mg-COD/mg-P) in the raw wastewater likely facilitated GAO proliferation in the sewer network.^63^ Detachment of bacteria from the biofilm grown in the sewer pipes possibly brings them into the sewage and influent. These GAOs are considered as potential competing immigrants coming with the influent wastewater to the AGS system. While GAOs compete with PAOs for COD, they typically do not impede the biological phosphorus removal process depending on the COD/P ratio, which indicates the presence of sufficient COD to support both microbial functional groups.^64^ As opposed to GAOs which had some of their species present in the AGS system due to immigration, all PAOs species had positive net-growth rates. Some of the GAOs that immigrated into the AGS system were probably effectively competed out by the PAOs. This could be either because they were less competitive in general or because they ended up in the floc fraction and did not get much substrate supply because they were at the top of the reactor during feeding; hence, they have negative net-growth rates. Furthermore, deterministic factors such as alternating anaerobic feast and famine strategy and the high food-to-microorganism feeding ratio minimize diffusion limitations and promote the growth of slow-growing PAOs.^56,57^ These PAOs were more enriched and expressed in SG and LG as compared with FL (Figure 4C), indicating the importance of granular sludge in AGS functionality. The FL samples contained only active species from fermentative PAO genera, specifically *Ca. Phosphoribacter* and *Tetrasphaera*, without any detectable activity from species of the genus *Ca. Accumulibacter* (Figure 6A). The positioning of flocculent sludge above the sludge bed fosters an environment favoring the fermentative PAO *Ca. Phosphoribacter*, driven by the presence of slowly biodegradable substrate, as the readily biodegradable substrate has already been consumed by the granular sludge situated at the bottom of the sludge bed.^6,64−66^*Tetrasphaera* and *Ca. Phosphoribacter* are commonly found in CAS and AGS systems receiving complex and slowly biodegradable wastewaters.^63^ Their metabolic versatility extends to a broader range of substrates, including sugars and amino acids, facilitating their utilization through fermentative processes compared to conventional PAOs like *Ca. Accumulibacter.*(67)

Our results exhibited a greater relative activity of some nitrifiers (AOB; *Nitrosomonas* sp. *RBC050*) in the influent and partially in FL (NOB; *Nitrotoga* sp*016721605*) as opposed to SG and LG (Figure 6). The higher relative activity in the influent samples is more likely because of the detachment of nitrifying bacteria from the biofilm grown in the sewer pipes sewer, which end up in the sewage due to the dispersal. Nitrifiers required a long SRT which is more available on biofilm systems of sewers. Previous studies also observed active nitrifiers present in the raw wastewater, seeding the downstream receiving bioreactor.^68,69^ This indicates that immigration plays a significant role in seeding the AGS bioreactor with important and beneficial microbes, such as nitrifiers and fermenters. In contrast to heterotrophic immigrants, which seem to be strongly influenced by deterministic selection, previous studies have suggested that nitrifiers tend to immigrate neutrally from sewers to activated sludge, and this could fully restore nitrification in reactors lacking nitrifying activity.^55^ Regarding the FL, aerobic nitrifiers demonstrate higher relative activity within smaller sized microbial aggregates, attributed to their larger surface area to volume ratio, resulting in increased aerobic biomass within these aggregates. Similarly, higher relative abundance of nitrifiers was detected in smaller sized microbial aggregates in the AGS and granular-based anammox bioprocess.^70,71^

In conclusion, our findings highlight the significant role of immigration in shaping and assembling the microbial community of the AGS system. Immigration contributes not only to the physical microbial population but also substantially to the system’s function. Furthermore, our study underscores the importance of the coexistence of microbial aggregates of different sizes for the functionality and benefits of the AGS system. These findings provide valuable insights for wastewater engineers, emphasizing the importance of considering influent microbial characterization when designing WWTPs to optimize the functionality and activity of the systems.

## Data Availability

Raw metagenomics and metatranscriptomics sequencing data and MAGs were deposited at the National Center for Biotechnology (NCBI) Sequence under accession number PRJNA1224817.

## Supporting Information Available

Appendix A: Methods of metagenomics and metatranscriptomic library preparation and squencing; photographs depicting the Ringsend Wastewater Treatment Plant; classifications of negative and positive net-growth-rate species calculated from metagenomics-based and metatranscriptomics-based mass balance; relative activity of negative and positive net-growth-rate species; differential expression analysis between aerobic and anaerobic conditions for varied-sized microbial aggregates; design and operational data for the AGS plant; measurements of total suspended solids, particle size distribution, and sludge volume index for MLSS, influent, effluent, and excess sludge samples; metagenomic Illumina short reads and Nanopore long reads sequencing processing data; and monthly average values of influent and effluent water quality parameters from the full-scale aerobic granular sludge unit in Ringsend, Ireland (PDF)
Appendix B: Properties, statistics, and taxonomy of 812 MAGs recovered from the process of assembly and binning (XLSX)
Appendix C: Relative abundances and expressions of the 812 MAGs across different samples (XLSX)
Appendix D: Metagenomics and metatranscriptomics mass balance calculations for samples collected on different dates (XLSX)
Appendix E: List of negative and positive net-growth-rate species in the AGS system based on metagenomics and metatranscriptomics mass balance calculations; and the relative activity of these species (XLSX)
