# Supplementary material for: Genome-Resolved Metatranscriptomics Provide Insights on Immigration Influence in Structuring Microbial Community Assembly of a Full-Scale Aerobic Granular Sludge Plant
Source: Environ Sci Technol. 2025 Mar 19;59(12):6126–41. doi: 10.1021/acs.est.4c14471 (PMC11966751; doi:10.1021/acs.est.4c14471)
Supplement: Supplementary file 1 — es4c14471_si_001.pdf [file es4c14471_si_001.pdf]

*For submission to Environmental Science and Technology (ES&T)*
**Revision of Manuscript (es-2024-14471g)**

# Genome-Resolved Metatranscriptomics Provide Insights on Immigration Influence in Structuring Microbial Community Assembly of a Full-Scale Aerobic Granular Sludge Plant

**A.Y.A. Mohamed [a], Laurence Gill [a], Alejandro Monleon [a], Mario Pronk [b], Mark van Loosdrecht [b], Pascal E. Saikaly [c,d], Muhammad Ali [a,*]**

[a] Department of Civil, Structural & Environmental Engineering, Trinity College Dublin, The University of Dublin, Dublin 2, Ireland

[b] Department of Biotechnology, Delft University of Technology, Delft 2629 HZ, The Netherlands

[c] Environmental Science and Engineering Program, Biological and Environmental Science and Engineering (BESE) Division, King Abdullah University of Science and Technology (KAUST), Thuwal 23955-6900, Saudi Arabia

[d] Water Desalination and Reuse Center, Biological and Environmental Science and Engineering (BESE) Division, King Abdullah University of Science and Technology (KAUST), Thuwal 23955-6900, Saudi Arabia

*Correspondence: M Ali, Department of Civil, Structural & Environmental Engineering, Trinity College Dublin, The University of Dublin, Dublin 2, Ireland. E-mail: Muhammad.ali@tcd.ie

Number of pages: 18
Number of figures: 7
Number of tables: 6

# 1. Supplementary materials and methods

## 1.1 Metagenomics and Metatranscriptomic library construction and sequencing

The steps of short reads-Metagenomics library constructions consist of DNA fragmentation and end preparation & dA-tailing, adapter ligation, cleanup, library amplification and final cleanup step. Adapter ligation was performed by forward Nextera adapter (3’): "AGATCGGAAGAGCACACGTCTGAACTCCAGTCAC"; and reverse Nextera adapter (5’): "AGATCGGAAGAGCGTCGTGTAGGGAAAGAGTGT". The steps of metatranscriptomic library constructions consist of deplete rRNA, fragment and denature RNA, synthesize first strand cDNA, synthesize second strand cDNA, adenylate 3′ends, ligate adapters, clean up libraries, amplify DNA fragments (PCR), perform second cleanup, and check libraries. The DNA and RNA concentration of the samples and quality of the library was measured and assessed with the Nanodrop (Thermo Fisher Scientific, Oregon, USA), Labchip GX (PerkinElmer, Waltham, Massachusetts, USA) and Qubit dsDNA HS Assay Kit and Qubit 4.0 Fluorometer (Invitrogen, Thermo Fisher Scientific, Oregon, USA). The integrity of the nucleic acids is assessed using agarose gel electrophoresis (Electrophoresis System: Tanon, EPS600; Electrophoresis Tank: Tiangen, HE-120). Sequencing of the metagenomic and Metatranscriptomic libraries was then performed on the Illumina NovaSeq 6000 platform, using paired-end 150 bp (PE150) sequencing, targeting a sequencing depth of 10 Gb per sample. A sequencing depth of 10 Gb and a PE read length of 150bp produced 300bp (150bp×2) per reading, 34 million of paired reads ((10 Gb)⁄(300 bp)), and 68 million of single reads((10Gb)⁄(150bp)).

The steps of Nanopore long reads-Metagenomics library constructions consist of : (1) Prepare 2 μg of HMW DNA; (2) Fragment the nucleic acid using a G-TUBE; (3) Perform DNA damage repair and end repair with A-tailing of the fragmented nucleic acid using NEBNext FFPE DNA Repair Mix and NEBNext Ultra II End Repair/dA-Tailing Module; (4) Add barcode sequences using the Non-Amplification Barcode Extension Kit 1-12 or Non-Amplification Barcode Extension Kit 13-24 and (5) Complete the ligation of sequencing adapters (adapter Y top: 5'-TTTTTTTTCCTGTACTTCGTTCAGTTACGTATTGCT-3’; adapter Y bottom: 5’-GCAATACGTAACTGAACGAAGTACAGG-3’) using the Non-Amplification Barcode Extension Kit 1-12 or Non-Amplification Barcode Extension Kit 13-24. The library quality control (QC) requires a Qubit concentration of >8 ng/μL to proceed with sequencing. The steps of library sequencing consist of: (1) Prepare Flow Cell Priming mix using the Sequencing Chip Preparation Kit (EXP-FLP002; Oxford Nanopore Technologies, Oxford, United Kingdom); (2) Prepare the on-board library using the Sequencing Auxiliary Expansion Kit (EXP-AUX001); (3) Using PromethION Flow Cells (FLO-PRO002; Oxford Nanopore Technologies, Oxford, United Kingdom) chips, run the MinKnow software on the PromethION48 (Oxford Nanopore Technologies, Oxford, United Kingdom) sequencer and start the sequencing. The default run time is 72 hours.

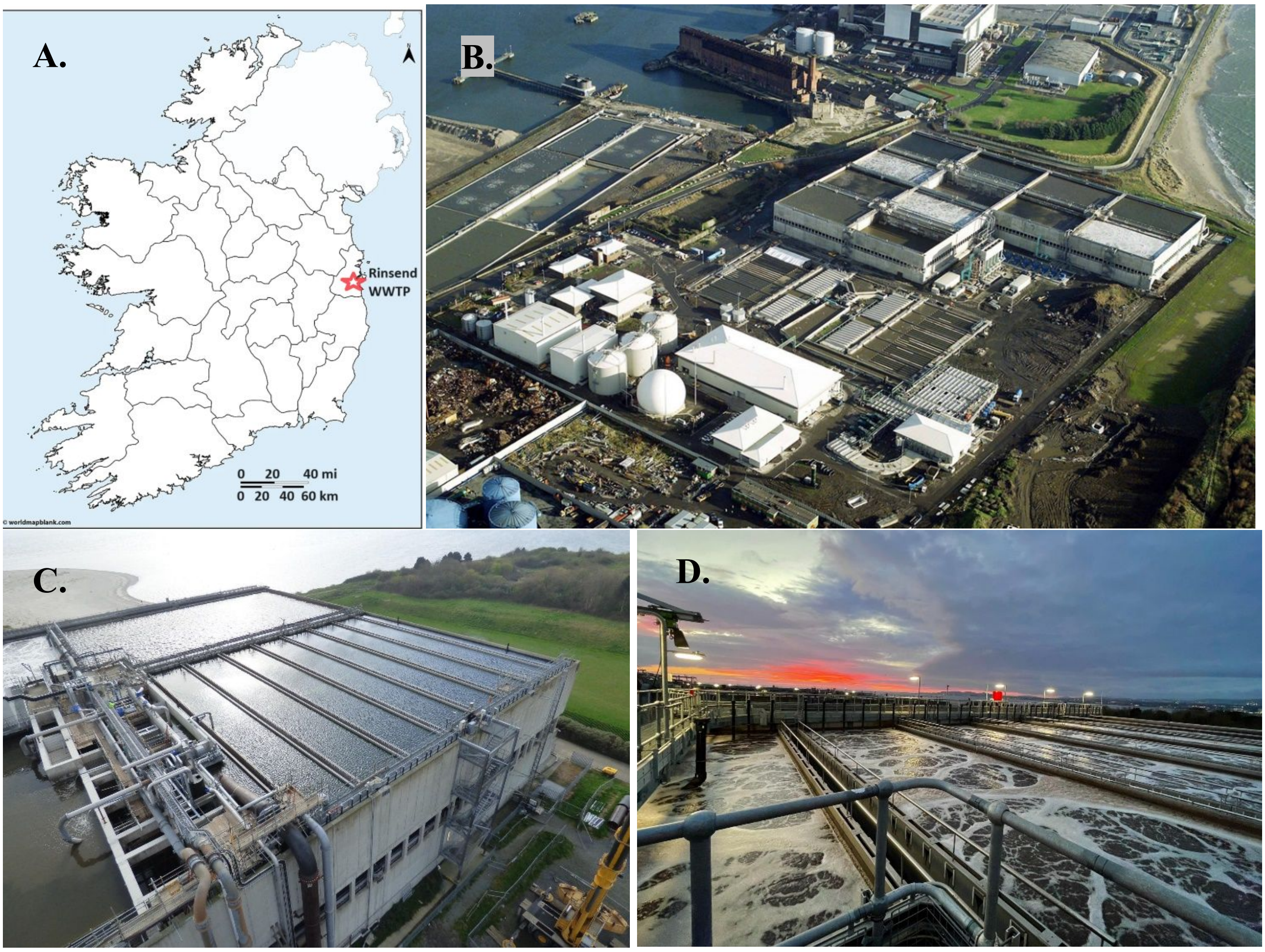


**Fig. S1** (A) Location of the Ringsend wastewater treatment plant in Ireland. (B) Aerial photograph showing the 24 pre-existing SBR tanks prior to retrofit constructed on two levels (12 SBR tanks per floor). (C) and (D) Aerial photograph showing the Nereda® aerobic granular sludge (AGS) reactors after SBR retrofitting.

**A-Negative-net-growth-rate species** **B-Positive-net-growth-rate species**

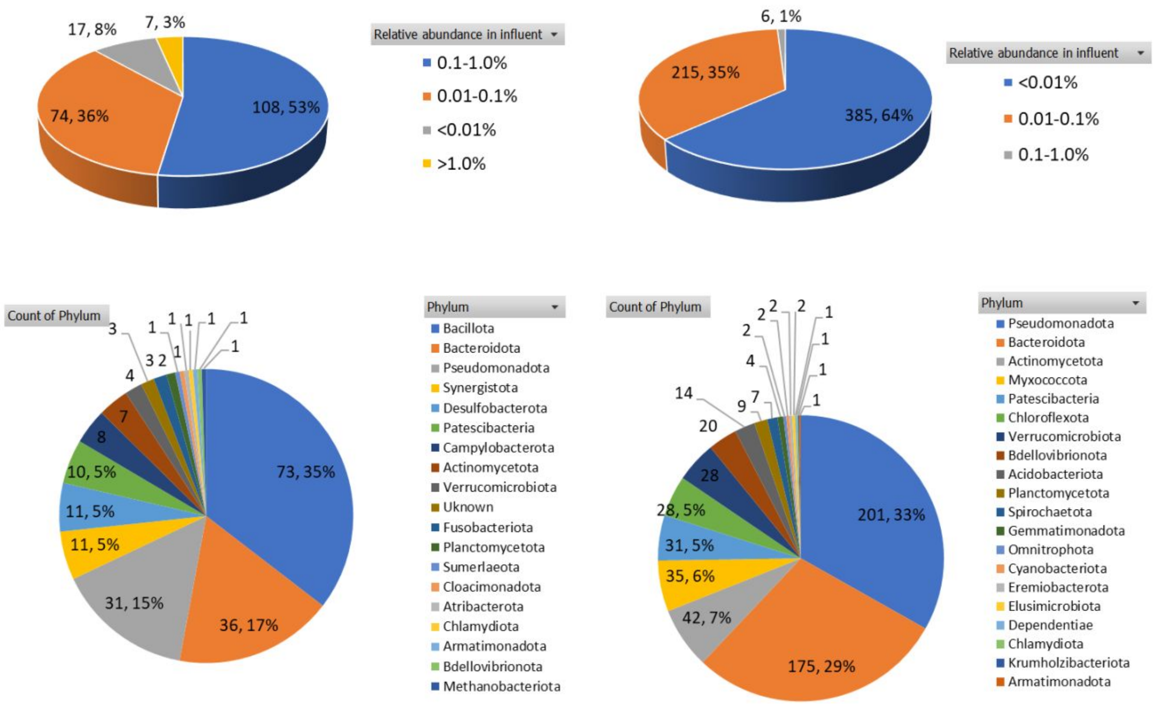


**Fig. S2** Classifications of negative (A) and positive (B) net-growth-rate species, calculated from metagenomics-based mass balance, into different categories: species relative abundance in influent (top); and Phylum classification (bottom).

**A-Negative-net-growth-rate species** **B-Positive-net-growth-rate species**

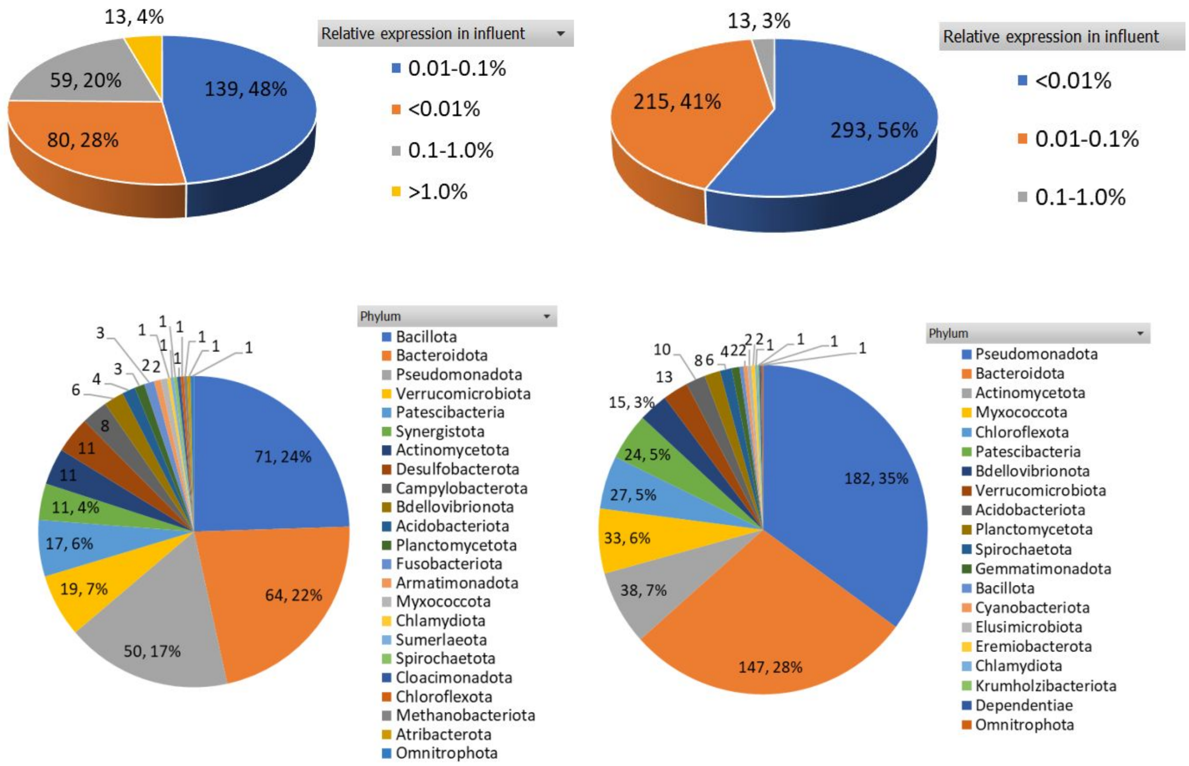


**Fig. S3** Classifications of negative (A) and positive (B) net-growth-rate species, calculated from metatranscriptomics-based mass balance, into different categories: species relative expression in influent (top); and Phylum classification (bottom).

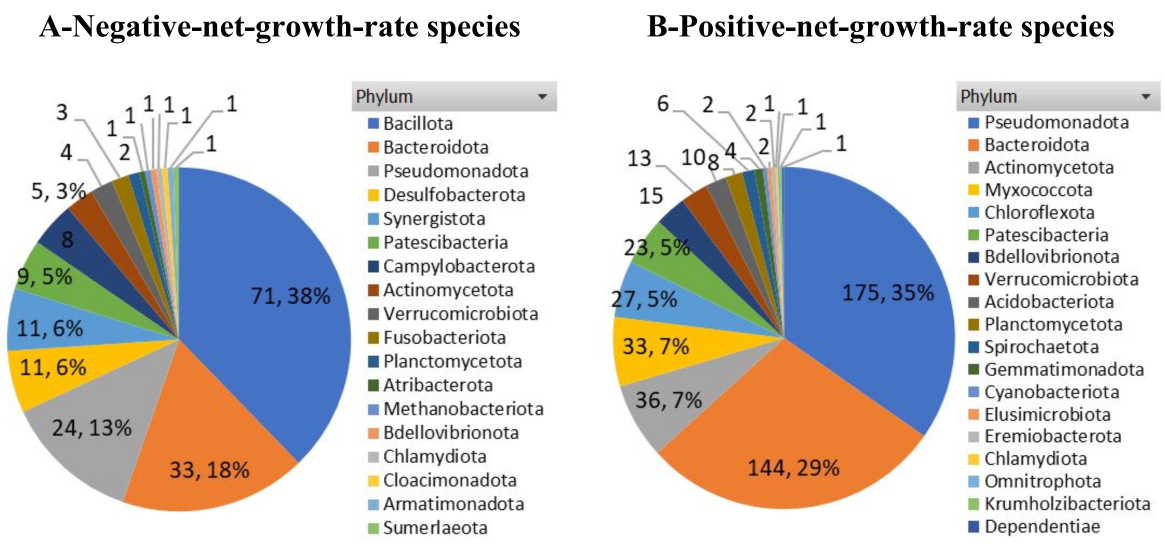


**Fig. S4** Phylum classifications of negative (A) and positive (B) net-growth-rate species, shared between metagenomics and metatranscriptomics-based mass balance calculations.

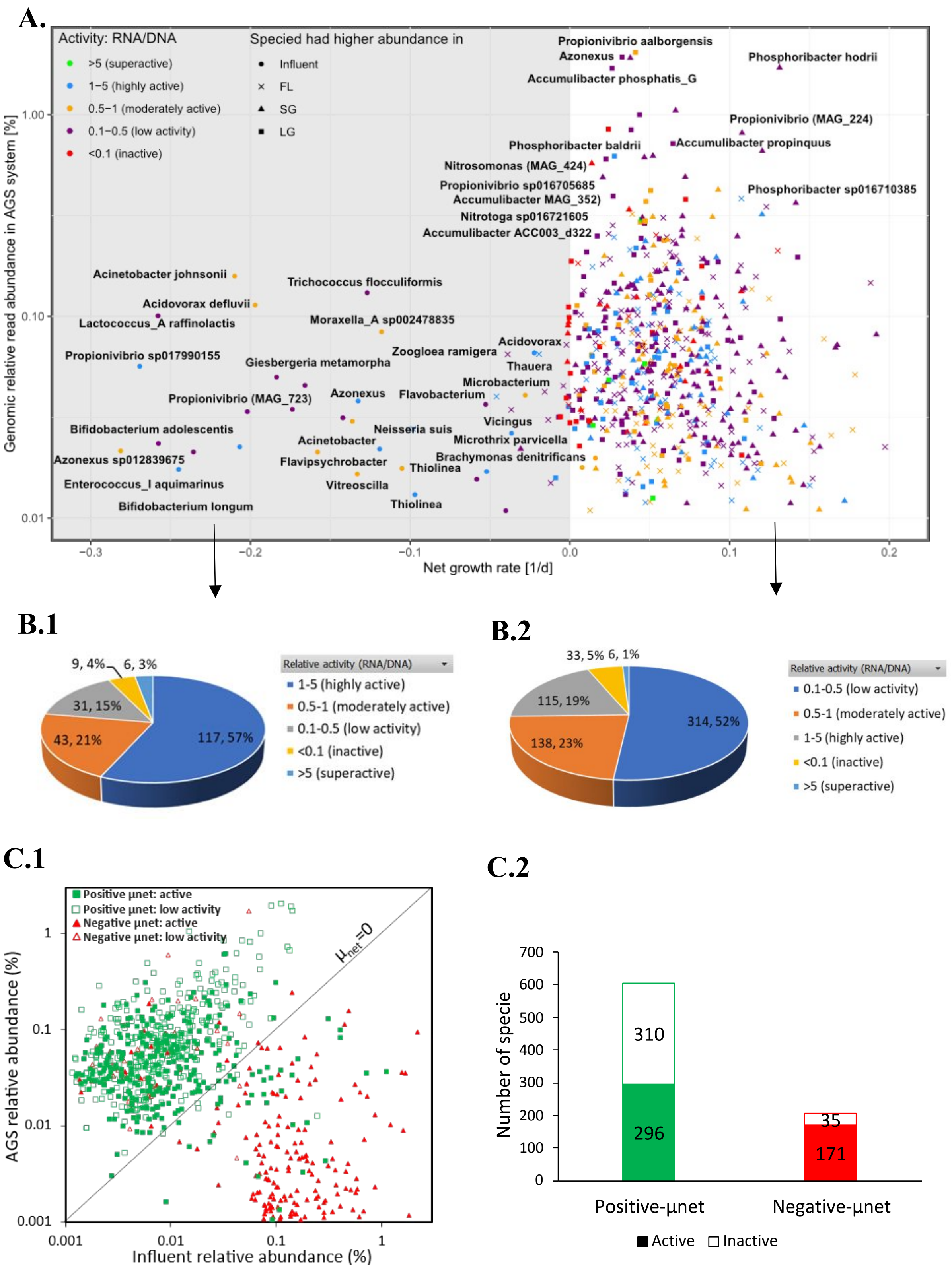


**Fig. S5** Relative activity of negative and positive net-growth-rate species calculated from metagenomics-based mass balance. A) Net growth rate vs. relative abundance (%) of species in AGS samples (25% FL, 53% SG, 22% LG). Species were coloured based on the relative activity into different categories. Species with growth rates < $-0.3$ d$^{-1}$ were excluded for plot

clarity. B) Numbers of species with different categories of relative activity for negative (B.1) and positive (B.2) growth groups. C) classification of positive (green square) and negative (red triangle) growth groups into species with higher (filled shape) and lower (empty shape) relative activity. C.1) Correlation of relative abundance in influent and AGS system. C.2) corresponding number of species with higher and lower relative activity for each group from plot (C.1). An RNA/DNA cutoff value of 0.434 **(Fig. 4A)** was used to delineate species with higher and lower relative activity.

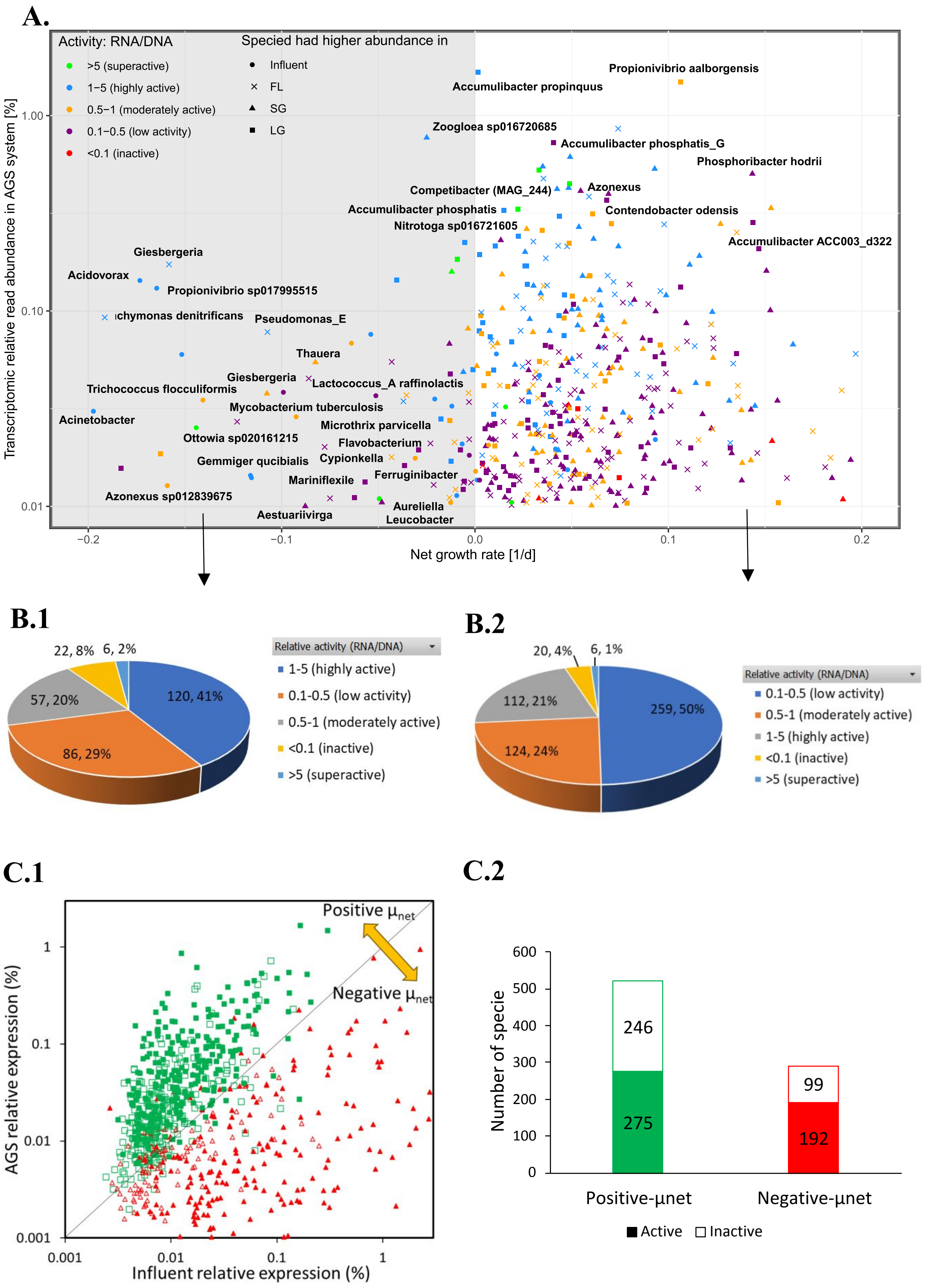


**Fig. S6** Relative activity of negative and positive net-growth-rate species calculated from metatranscriptomics-based mass balance. A) Net growth rate vs. relative expression (%) of species in AGS samples (25% FL, 53% SG, 22% LG). Species were coloured based on the relative activity into different categories. Species with growth rates < −0.3 $d^{-1}$ were excluded

for plot clarity. B) Numbers of species with different categories of relative activity for negative (B.1) and positive (B.2) growth groups. C) classification of positive (green square) and negative (red triangle) growth groups into species with higher (filled shape) and lower (empty shape) relative activity. C.1) Correlation of relative expression in influent and AGS system. C.2) corresponding number of species with higher and lower relative activity for each group from plot (C.1). An RNA/DNA cutoff value of 0.434 **(Fig. 4A)** was used to delineate species with higher and lower relative activity.

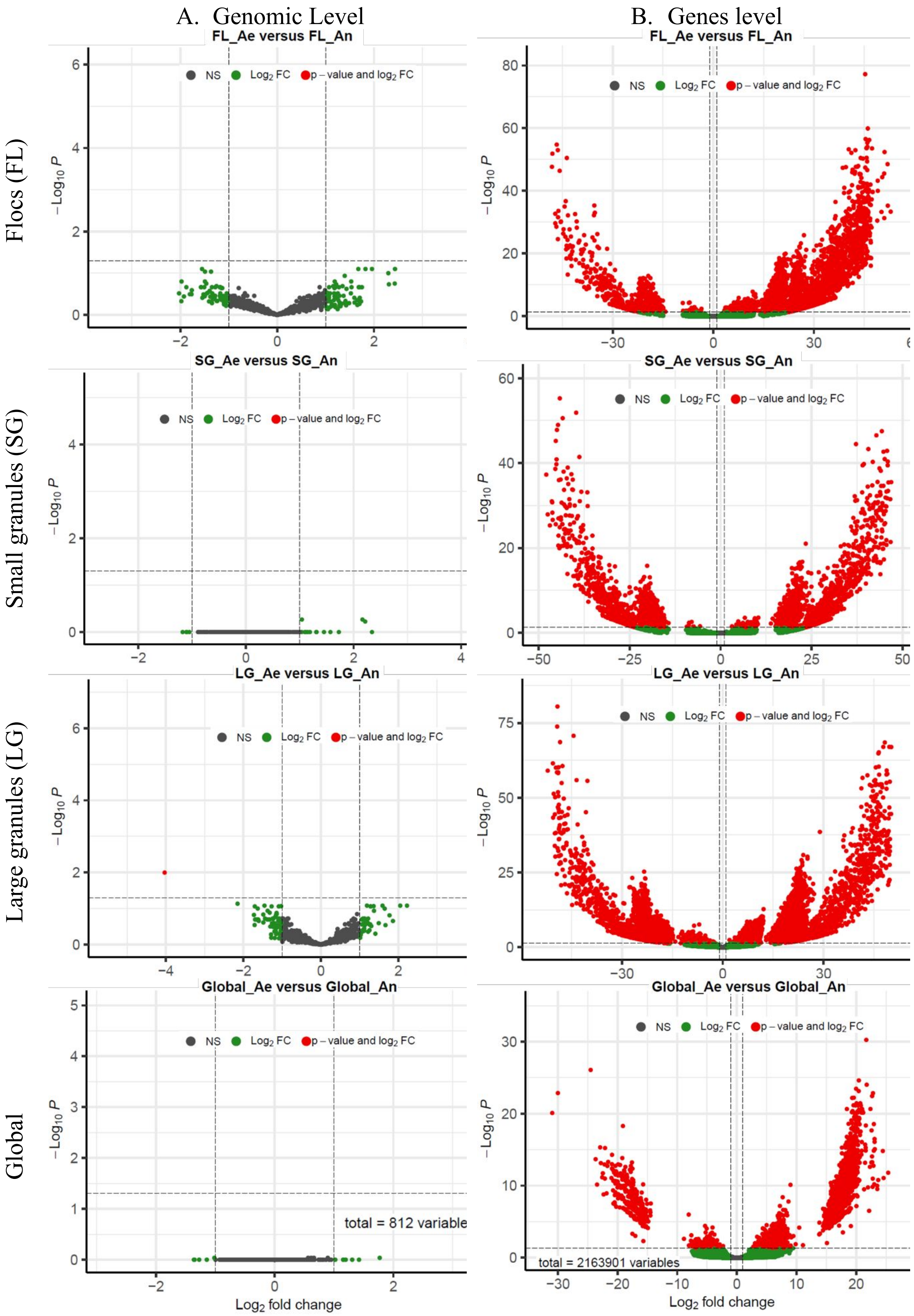


**Fig. S7** EnhancedVolcano plots showing differential expression analysis between aerobic (Ae) and anaerobic (An) conditions for varied-sized microbial aggregates (flocs (FL), small granules (SG), large granule (LG), and global) using DESeq2. A) based on genomic level

(812 genomes); B) based on genes level (2163901 genes). Species were coloured based on the level of significance and degrees of variation; either as absolute log2 fold change |log2 FC| ≥ 1 or P-value < 5% or meeting both |log2 FC| ≥ 1 and P-value < 5%, or meeting none of the previous condition as non-significant (NS). Genomes and genes with negative log2 FC indicate they were downregulated (less active) aerobically, while positive log2 FC values indicate they were upregulated (more active) aerobically.

**Table S1** Design and operational data for the AGS plant

| Parameter | Value | Unit |
|---|---|---|
| **Flows and capacity** | | |
| Population Equivalent | 1.7 million | PE |
| Organic Capacity - Design / As Constructed | 1,640,000 | PE |
| Organic Capacity - Current loading - peak week load | 2,278,887 | PE |
| DWF | 4.6 | $m^3/s$ |
| Average flow | 5.7 | $m^3/s$ |
| Full flow to Treatment (Design Flow) | 11.1 | $m^3/s$ |
| Stormwater Flow | 11.5 | $m^3/s$ |
| Peak flow to Site | 22.6 | $m^3/s$ |
| Peak Hydraulic Capacity- As Constructed | 959,040 | $m^3$/day |
| DWF to the Treatment Plant | 274,076 | $m^3$/day |
| Current Hydraulic Loading - annual max | 832,269 | $m^3$/day |
| Average Hydraulic loading to the Treatment Plant | 458,641 | $m^3$/day |
| Average DWF | 492480 | $m^3/d$ |
| Design concentration of mixed liquor suspended solids (MLSS) | 4.0 | $Kg/m^3$ |
| **Reactor size** | | |
| No. Reactor Basins | 24 | |
| Length | 52 | m |
| Width | 39 | m |
| TWL | 6.9 | m |
| BWL | 4.9 | m |
| Volume of one SBR | 13,993 | $m^3$ |
| Total Process Volume | 335,832 | $m^3$ |
| Aeration Devices | Fine bubble diffused air | |
| **Design Sludge Loads for 24 SBR Tanks** | | |
| Primary Sludge production @ 3%ds | 53,612 | kg/d |
| Primary Sludge flow | 1,787 | $m^3/d$ |
| Secondary Sludge production @ 0.85%ds | 52,000 | kg/d |
| Secondary Sludge flow | 6,118 | $m^3/d$ |
| Combined Sludge Load @ 1.34%ds | 105,612 | kg/d |
| Combined Sludge flow | 7,905 | $m^3/d$ |

**Table S2** Measurements of total suspended solids (TSS), particle size distribution (PSD) and sludge volume index (SVI) for MLSS, influent, effluent, and excess sludge samples collected from Nereda AGS reactor.

| Sample | phase | size | TSS (mg/l) | PSD (%) | SVI-5 min (ml/g) | SVI-30 min (ml/g) |
|---|---|---|---|---|---|---|
| MLSS | Aerobic | LG | 825.9 | 21.9 | | |
| MLSS | Aerobic | SG | 1990.0 | 52.8 | | |
| MLSS | Aerobic | Fl | 951.9 | 25.3 | | |
| MLSS | Aerobic | Mix-calc | 3767.8 | | | |
| MLSS | Aerobic | Mix-real | 3860.0 | | 103.6 | 59.6 |
| MLSS | Anaerobic | LG | 211.1 | 7.0 | | |
| MLSS | Anaerobic | SG | 2114.3 | 69.9 | | |
| MLSS | Anaerobic | Fl | 700.0 | 23.1 | | |
| MLSS | Anaerobic | Mix-calc | 3025.4 | | | |
| MLSS | Anaerobic | Mix-real | 3616.7 | | | |
| Excess sludge | | LG | 123.08 | 5.0 | | |
| Excess sludge | | SG | 1107.72 | 45.0 | | |
| Excess sludge | | Fl | 1230.8 | 50.0 | | |
| Excess sludge | | Mix-calc | 2461.6 | | | |
| Excess sludge | | Mix-real | 2571.4 | | | |
| Influent | | | 128.0 | | | |
| Effluent | | | 61.8 | | | |

**Table S3** Metagenomic Illumina short reads sequencing processing data/statistics and quality

| Sample - ID | Sample type | Raw reads | Raw Data (Gb) | Clean reads (%) | Q20(%) | Binned (mapped) reads | Binned (mapped) reads(%) |
|---|---|---|---|---|---|---|---|
| G-1 | FL_R1 | 70198552 | 10.53 | 97.82 | 97.13 | 48072683 | 68.48 |
| G-2 | FL_R2 | 70807148 | 10.59 | 98.37 | 99.87 | 44882668 | 63.39 |
| G-3 | FL_R3 | 70502850 | 10.56 | 98.81 | 98.5 | 51872357 | 73.57 |
| G-4 | SG_R1 | 71822878 | 10.77 | 99.19 | 97.13 | 51486422 | 71.69 |
| G-5 | SG_R2 | 79188310 | 11.86 | 97.97 | 99.85 | 56296834 | 71.09 |
| G-6 | SG_R3 | 75505594 | 11.315 | 98.79 | 98.49 | 54574068 | 72.28 |
| G-7 | LG_R1 | 72338874 | 10.85 | 99.14 | 97.39 | 49260626 | 68.10 |
| G-8 | LG_R2 | 82267552 | 12.32 | 98.86 | 99.88 | 53192991 | 64.66 |
| G-9 | LG_R3 | 77303213 | 11.585 | 98.84 | 98.635 | 55299265 | 71.54 |
| G-10 | IN_R1 | 69181294 | 10.38 | 98.29 | 97.41 | 30400491 | 43.94 |
| G-11 | IN_R2 | 75172152 | 11 | 98.2 | 99.91 | 44385743 | 59.05 |
| G-12 | IN_R3 | 89544476 | 11.28 | 99.17 | 99.53 | 50291833 | 56.16 |
| G-13 | EF_R1 | 69568820 | 10.44 | 98.55 | 97.55 | 52641548 | 75.67 |
| G-14 | EF_R2 | 75172152 | 11.24 | 98.52 | 99.89 | 52892118 | 70.36 |
| G-15 | EF_R3 | 89544476 | 13.41 | 97.9 | 99.78 | 68735997 | 76.76 |
| G-16 | ES_R1 | 70009866 | 10.5 | 98.4 | 97.43 | 54782531 | 78.25 |
| G-17 | EF_R2 | 75172152 | 11.24 | 98.3 | 99.89 | 53414521 | 71.06 |
| G-18 | ES_R3 | 79293598 | 11.76 | 99.1 | 99.88 | 67750987 | 85.44 |

**Table S4** Metatranscriptomics Illumina short reads sequencing processing data/statistics and quality.

| Sample -ID | Sample type | Raw reads | Raw Data (Gb) | Clean reads (%) | Binned (mapped) reads | Binned (mapped) reads(%) |
|---|---|---|---|---|---|---|
| T-1 | FL_An_R1 | 75283978 | 11.29 | 99.6 | 39786304 | 52.85 |
| T-2 | FL_An_R2 | 70412248 | 10.56 | 99.6 | 27243727 | 38.69 |
| T-3 | FL_An_R3 | 66957960 | 10.04 | 99.6 | 25035578 | 37.39 |
| T-4 | FL_Ae_R1 | 65874614 | 9.88 | 99.6 | 28884821 | 43.85 |
| T-5 | FL_Ae_R2 | 83005932 | 12.45 | 99.6 | 40229881 | 48.47 |
| T-6 | FL_Ae_R3 | 69749762 | 10.46 | 99.6 | 46913104 | 67.26 |
| T-7 | SG_An_R1 | 75455404 | 11.32 | 99.6 | 40966369 | 54.29 |
| T-8 | SG_An_R2 | 69290294 | 10.39 | 99.6 | 51117319 | 73.77 |
| T-9 | SG_An_R3 | 66950962 | 10.04 | 99.6 | 43156425 | 64.46 |
| T-10 | SG_Ae_R1 | 68888140 | 10.33 | 99.6 | 36371805 | 52.80 |
| T-11 | SG_Ae_R2 | 70115436 | 10.52 | 99.6 | 47047967 | 67.10 |
| T-12 | SG_Ae_R3 | 70524314 | 10.58 | 99.6 | 47919758 | 67.95 |
| T-13 | LG_An_R1 | 70342148 | 10.55 | 99.6 | 34927663 | 49.65 |
| T-14 | LG_An_R2 | 66952572 | 10.04 | 99.6 | 38797731 | 57.95 |
| T-15 | LG_An_R3 | 74905598 | 11.24 | 99.6 | 58437996 | 78.02 |
| T-16 | LG_Ae_R1 | 69023326 | 10.35 | 99.6 | 45975243 | 66.61 |
| T-17 | LG_Ae_R2 | 78250502 | 11.74 | 99.6 | 41954747 | 53.62 |
| T-18 | LG_Ae_R3 | 68777440 | 10.32 | 99.6 | 48724693 | 70.84 |
| T-19 | IN_R1 | 68704788 | 10.31 | 99.6 | 33516572 | 48.78 |
| T-20 | IN_R2 | 71042740 | 10.66 | 99.6 | 46407784 | 65.32 |
| T-21 | IN_R3 | 69234984 | 10.39 | 99.6 | 46027739 | 66.48 |
| T-22 | EF_R1 | 74501444 | 11.18 | 99.6 | 34726003 | 46.61 |
| T-23 | EF_R2 | 73244042 | 10.99 | 99.6 | 48528901 | 66.26 |
| T-24 | EF_R3 | 68694116 | 10.3 | 99.6 | 45635430 | 66.43 |
| T-25 | ES_R1 | 70174370 | 10.53 | 99.6 | 54948227 | 78.30 |
| T-26 | ES_R2 | 69265544 | 10.39 | 99.6 | 51694360 | 74.63 |
| T-27 | ES_R3 | 68886388 | 10.33 | 99.6 | 52829078 | 76.69 |

**Table S5** Metagenomic Nanopore long reads sequencing processing data/statistics and quality.

| Description | Flocs | Small Granule | Large Granule | Excess Sludge |
|---|---|---|---|---|
| Raw reads | 5024474 | 2267806 | 2215975 | 3445528 |
| Raw reads (Gb) | 17.07 | 11.07 | 10.73 | 14.68 |
| Clean reads | 4107639 | 2031106 | 1960759 | 2989907 |
| Clean reads (Gb) | 15.91 | 10.61 | 10.16 | 13.9 |
| Clean N50 Length | 4934 | 6882 | 6896 | 6031 |
| Clean N90 Length | 1916 | 2620 | 2558 | 2338 |
| Clean Mean Length | 3872 | 5223 | 5181 | 4649 |
| Clean Max Length | 174318 | 96519 | 95746 | 148611 |
| Clean Mean Qual | 10 | 10 | 10 | 10 |

**Table S6** Monthly averages of the influent and effluent from the full-scale aerobic granular sludge unit in Ringsend, Ireland.

| **Parameter** | **Influent (Average)** | **Effluent (Average)** |
|---|---|---|
| $BOD_5$ (mg/L) | 224 | 16±4 |
| COD (mg/L) | 506 | 66±9 |
| TN (mg/L) | 49.4 | 7±1 |
| $NH_4^+$-N (mg/L) | 39 | 3±1.5 |
| TP (mg/L) | 6.7 | 0.81±0.12 |
| $PO_4^{3-}$-P (mg/L) | 4.4 | 0.33±0.08 |

Influent data obtained from 2022 annual report:
https://www.water.ie/sites/default/files/docs/aers/2022/D0034-01_2022_AER.pdf
